# Goldilocks at the dawn of complex life: mountains might have damaged Ediacaran–Cambrian ecosystems and prompted an early Cambrian greenhouse world

**DOI:** 10.1038/s41598-021-99526-z

**Published:** 2021-10-08

**Authors:** Fabricio Caxito, Cristiano Lana, Robert Frei, Gabriel J. Uhlein, Alcides N. Sial, Elton L. Dantas, André G. Pinto, Filippe C. Campos, Paulo Galvão, Lucas V. Warren, Juliana Okubo, Carlos E. Ganade

**Affiliations:** 1 CPMTC Research Center, Universidade Federal de Minas Gerais, Belo Horizonte, MG 31270-901 Brazil; 2 Departamento de Geologia, Universidade Federal de Ouro Preto, Ouro Preto, MG 35400-000 Brazil; 3 Department of Geoscience and Natural Resource Management, University of Copenhagen, Øster Voldgade 10, 1350 Copenhagen, Denmark; 4 NEG-LABISE, Universidade Federal de Pernambuco, Recife, PE 50740-530 Brazil; 5 Laboratório de Estudos Geodinâmicos, Geocronológicos E Ambientais, Universidade de Brasília, Brasília, DF 70910-900 Brazil; 6 Department of Geology, São Paulo State University, Rio Claro, SP 13506-900 Brazil; 7 Geological Survey of Brazil – CPRM, Rio de Janeiro, RJ 22290-255 Brazil

**Keywords:** Geochemistry, Geology

## Abstract

We combine U–Pb in-situ carbonate dating, elemental and isotope constraints to calibrate the synergy of integrated mountain-basin evolution in western Gondwana. We show that deposition of the Bambuí Group coincides with closure of the Goiás-Pharusian (630–600 Ma) and Adamastor (585–530 Ma) oceans. Metazoans thrived for a brief moment of balanced redox and nutrient conditions. This was followed, however, by closure of the Clymene ocean (540–500 Ma), eventually landlocking the basin. This hindered seawater renewal and led to uncontrolled nutrient input, shallowing of the redoxcline and anoxic incursions, fueling positive productivity feedbacks and preventing the development of typical Ediacaran–Cambrian ecosystems. Thus, mountains provide the conditions, such as oxygen and nutrients, but may also preclude life development if basins become too restricted, characterizing a Goldilocks or optimal level effect. During the late Neoproterozoic-Cambrian fan-like transition from Rodinia to Gondwana, the newborn marginal basins of Laurentia, Baltica and Siberia remained open to the global sea, while intracontinental basins of Gondwana became progressively landlocked. The extent to which basin restriction might have affected the global carbon cycle and climate, e.g. through the input of gases such as methane that could eventually have collaborated to an early Cambrian greenhouse world, needs to be further considered.

## Introduction

Evidence for single-celled organisms dates far back to the Eoarchean or even to the Hadean^1^, but complex life only became widespread in Earth’s oceans during the late Ediacaran (ca. 575–560 Ma^2^), broadly coincident with some of the planet’s most extreme climatic^3^, tectonic^4,5^ and redox^6^ variations. This broad coincidence led to proposals of intricate feedback loops between all of those spheres, especially regarding the influence of mountain belts over adjoining complex-life bearing basins^7,8^. To sustain complex life, the availability of nutrients and oxygen are considered as the key limiting factors^7–10^, both of which could be readily available through enhanced weathering of uplifted areas^7,8,11^. Weathering of mountain chains consumes CO_2_, the main greenhouse gas, thus modulating Earth’s climate. Besides, the high influx of sediments into adjacent foreland basins causes high rates of organic carbon burial that cannot back-react^2^. The net result is a growth of free oxygen levels in Earth’s atmosphere, which in turn may prompt evolution of complex life forms, fueling back the spiraled feedback loops^8^. Accordingly, the development of extensive mountain chains was proposed as an important factor in providing the necessary oxygen and nutrients for early metazoan evolution^7,8,11^. However, other possible, even deleterious effects of mountain ranges in the biogeochemical conditions of adjacent life-supporting sedimentary basins are not yet fully considered.

Although plate tectonics under regimes of shallower and hotter subduction might have operated since the Mesoarchean (“Proterozoic-style plate tectonics”^4^), a change to modern-style plate tectonics characterized by deep subduction and colder thermal gradients apparently occurred in the Neoproterozoic, as suggested by the global distribution of ophiolites, blueschists and UHP (Ultra-High Pressure) rocks^4,5^, especially in the Pan-African/Brasiliano orogens that formed during the amalgamation of Gondwana^7,8,11,12^. This is a consequence of secular cooling of the mantle, which by the end of the Neoproterozoic might have cooled sufficiently to allow widespread cold and dense lithosphere slabs to collapse into the underlying asthenosphere without loosing its coherence. Deep subduction of felsic continental crust and colder geotherms led to continental collision zones with deep roots and lower density, resulting in significant relief generated by isostatic rebound^13^. Thus, the inception of modern-style plate tectonics produced large mountain belts up to thousands of km long and topographically higher than pre-Neoproterozoic orogens. High relief in mountainous areas can be maintained for at least ca. 40 Myr after the onset of continental collision^14^, providing detritus for long-lived adjacent sedimentary basins over broad timescales.

As an important outcome, extensive Ediacaran–Cambrian sedimentary basins developed throughout Gondwana, fed by denudation of the recently uplifted mountain belts. Late Ediacaran to early Cambrian metazoan biota that appears for the first time in the stratigraphic record has been described^15–17^ in all of the basins that sourced the Pan-African/Brasiliano orogens as main provenance areas.

To test how interlinked the development of mountain belts and metazoan-bearing sedimentary basins were in western Gondwana, we performed in-situ Laser Ablation-Induced Coupled Plasma (LA-ICPMS) determinations of U–Pb, Sr isotope and trace element data on samples from distinct carbonate levels in the Ediacaran–Cambrian Bambuí Group of east-central Brazil, along with novel stepwise Pb leaching mineral dating and Sr, C and O isotope data. We produced a comprehensive compilation of C, Sr, Nd isotope and detrital zircon data for different sections of this basin, and discuss other available proxies in light of the integrated framework of orogen-basin evolution proposed here. The Bambuí Group is ideal for testing the hypothesis of integrated metazoan-mountain evolution as it is located at the core of western Gondwana and surrounded by collisional mountain belts developed diachronously around the São Francisco paleocontinent^18^, between 630 and 600 Ma (Brasília Orogen to the southwest)^18^, 585–530 Ma (Araçuaí-Ribeira Orogen to the east)^19^ and 540–500 Ma (Araguaia-Paraguay-Pampean Orogen to the northwest)^20^. The goal is to investigate the influence of the uplifting mountains in the sedimentary and biological record of the first metazoan-bearing basins by tectonic restriction of epeiric seas and changes in continentally-derived nutrient influx through time. We argue that progressive basin restriction by the surrounding mountains might have damaged the conditions for complex life development and discuss the possible global outcome of widespread basin restriction in Gondwana and its effects on global biogeochemical cycles.

### Geological context

**Figure 1:**
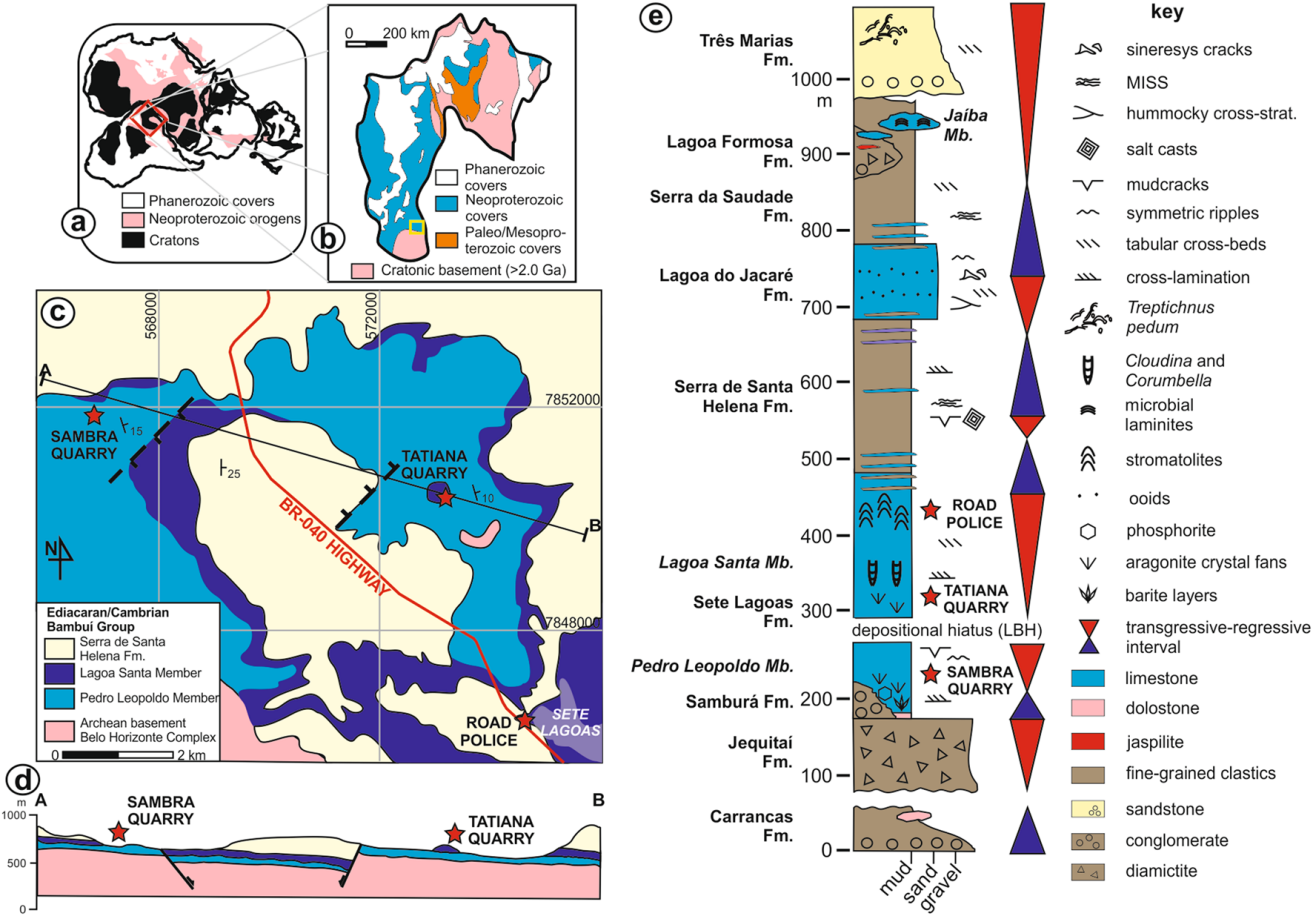
Location and stratigraphy of the Bambuí Group. (**a**) Location of the Bambuí Group in western Gondwana and (**b**) in the São Francisco craton. In (**c**) and (**d**), map and section of the studied area, respectively. In (**e**), schematic section of the Bambuí Group, compiled from^23,25^. The maps were created using Corel Draw Graphics Suite 2018 (http://www.coreldraw.com) and a Huion Kamvas GS1331B pen display (http://www.huion.com).

Deposition of the Bambuí Group spanned the whole Ediacaran and lower Cambrian over the São Francisco paleocontinent^21–23^ (Fig. 1a–d), comprising a mixed carbonate-siliciclastic succession. The complete stratigraphic package is illustrated in the schematic lithostratigraphic column of Fig. 1e. Glacial diamictite at the base of the group rests atop striated pavements^24^ and is probably related to the global Marinoan glaciation^21,25^. The overlying Sete Lagoas Formation represents the basal carbonate succession of the Bambuí Group and is subdivided into two members (Fig. 1c,d).

The lower Pedro Leopoldo Member covers the glacial deposits or onlaps the crystalline basement and comprises a typical early Ediacaran cap carbonate succession. At the base, a meter-thick patchy cap dolostone unit shows decreasing-upwards δ^13^C_carb_ from − 3.2‰ down to − 6.5‰ and associated δ^18^O at − 5‰^21^ (all values reported as compared to VPDB). The cap dolostone is succeeded by up to a couple hundred meters-thick limestone containing pseudomorphs of calcite after original aragonite crystal fans with negative δ^13^C_carb_^21,23,25^. Phosphorite deposits^26^, apatitic cements^27^ and centimetric barite layers with a characteristic Δ^17^O anomaly^28^, probably caused by perturbations in the ozone layer due to excess CO_2_ accumulated during the Marinoan glaciation, are locally recognized^27,28^. Cr isotope and geochemical data (negative Ce anomalies, low Th/U ratios, Mo and U contents, and Fe speciation data^25,29^) suggest pulsed oxygenation of the post-glacial ocean due to meltwater contribution^25^.

The top of the Pedro Leopoldo cap carbonate succession is marked by a depositional hiatus or condensed section, recognized in both seismic and isotopic breaks^21,30^. Although some suggestions that this surface might represent an erosional unconformity were put forward, no convincing field evidence other than dissolution features, tepees, mud cracks, dolomitization and other facies changes, as well as subtle variations in regional dip^30^ have yet been described, so it is safer to assume this interval as a depositional hiatus, here defined as the Lower Bambuí Hiatus—LBH. Above the LBH lies the couple hundred meters-thick Lagoa Santa Member, comprising a second crystal-fan-bearing limestone level superimposed by laminar and columnar stromatolites and thrombolites with δ^13^C_carb_ at ca. 0‰. This intermediate succession contains some putative trace fossils and sparse, loosely packed *Cloudina* sp.^16^ shells and *Corumbella werneri*^17^ fragments^15^. The δ^13^C_carb_ values rise quickly upwards to >  + 10‰, reaching extreme values of ca. + 16‰^21,23^ and the macrofossil content virtually disappears. These δ^13^C_carb_ values are anomalously high when compared to Ediacaran global curves and persist upsection for around 350 m, spanning through the siltstone-dominated Serra de Santa Helena Formation and dark storm-related limestone of the Lagoa do Jacaré Formation, defining the Middle Bambuí Excursion (MIBE)^23^.

Above the *Cloudina*-bearing interval, the remainder of the Bambuí Group is mostly devoid of macrofossils and anoxic conditions prevailed throughout the water column, as shown by isotopic, elemental and Fe speciation data^23,29,31,32^. The widespread anoxic conditions might have been predominant up to the lower Cambrian Series 2, according to U–Pb zircon dating of a tuff layer of the Serra da Saudade Formation at the upper part of the Bambuí Group at 520.2 ± 5.3 Ma^33^. Somehow, after a pre-MIBE bloom of metazoans, geochemical conditions became hazardous to complex organisms, hindering evolution and preventing an expected rise of typical macrofaunal biota as observed in other Ediacaran–Cambrian basins worldwide^29^.

## Results

**Figure 2:**
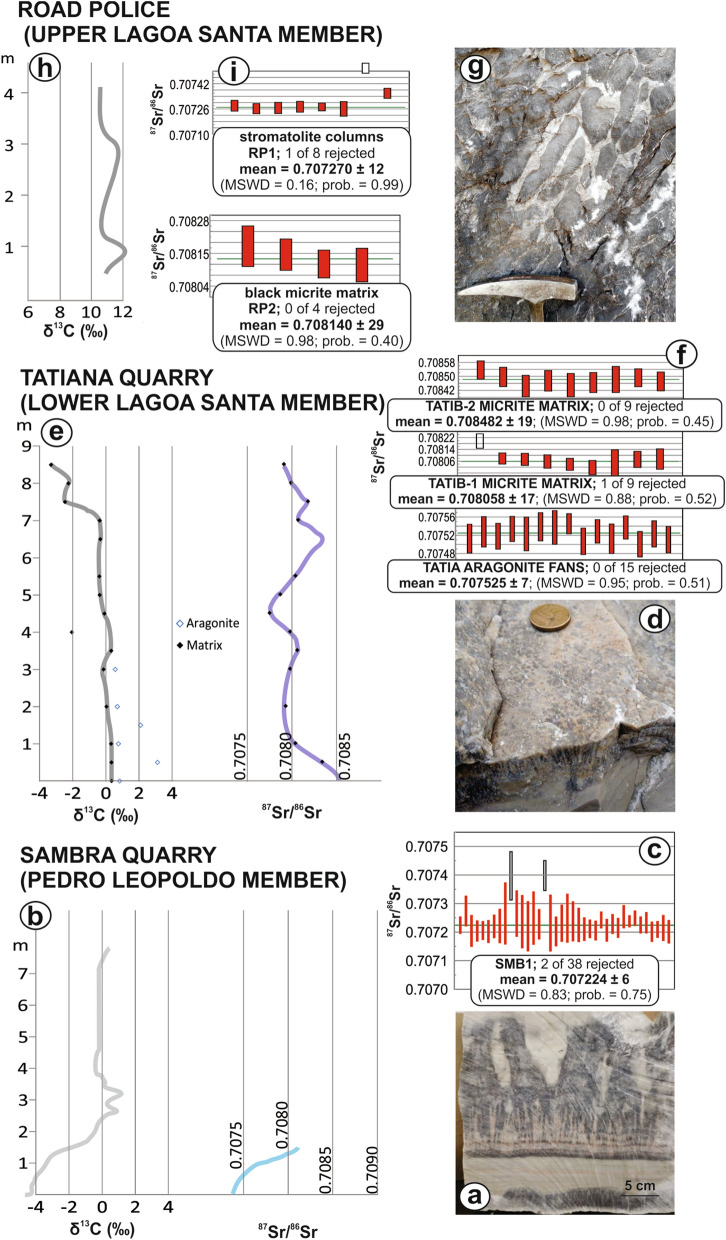
Field pictures and isotopic results of the studied rock samples. Calcite pseudomorphs after aragonite-fan bearing carbonates of the Pedro Leopoldo Member (**a**) with typical cap carbonate δ^13^C_carb_ (**b**) and early Ediacaran ^87^Sr/^86^Sr at ca. 0.7072 (**c**); Calcite pseudomorphs after aragonite-fan bearing carbonates of the Lagoa Santa Member (**d**) with near-zero δ^13^C_carb_ (**e**) and late Ediacaran ^87^Sr/^86^Sr around 0.7080 (**f**); Dark stromatolitic carbonates of the topmost Lagoa Santa Member (**g**) with δ^13^C_carb_ > 10‰ corresponding to the MIBE (**h**) and ^87^Sr/^86^Sr back to values at ca. 0.7072 (**i**). Whole-rock values of δ^13^C_carb_ and ^87^Sr/^86^Sr for the Tatiana Quarry (**e**) and all in-situ ^87^Sr/^86^Sr values are from this work; whole-rock values of δ^13^C_carb_ and ^87^Sr/^86^Sr for the Sambra (**b**) and Road Police (**h**) sections are compiled from the literature^34,35^. Charts were created using Microsoft Excel Professional Plus 2016 (http://www.microsoft.com), the Isoplot 3.6 Visual Basic Add-in freeware by Ken Ludwig, Berkeley Geochronology Center (http://www.bgc.org/isoplot) and Corel Draw Graphics Suite 2018 (http://www.coreldraw.com).

**Figure 3:**
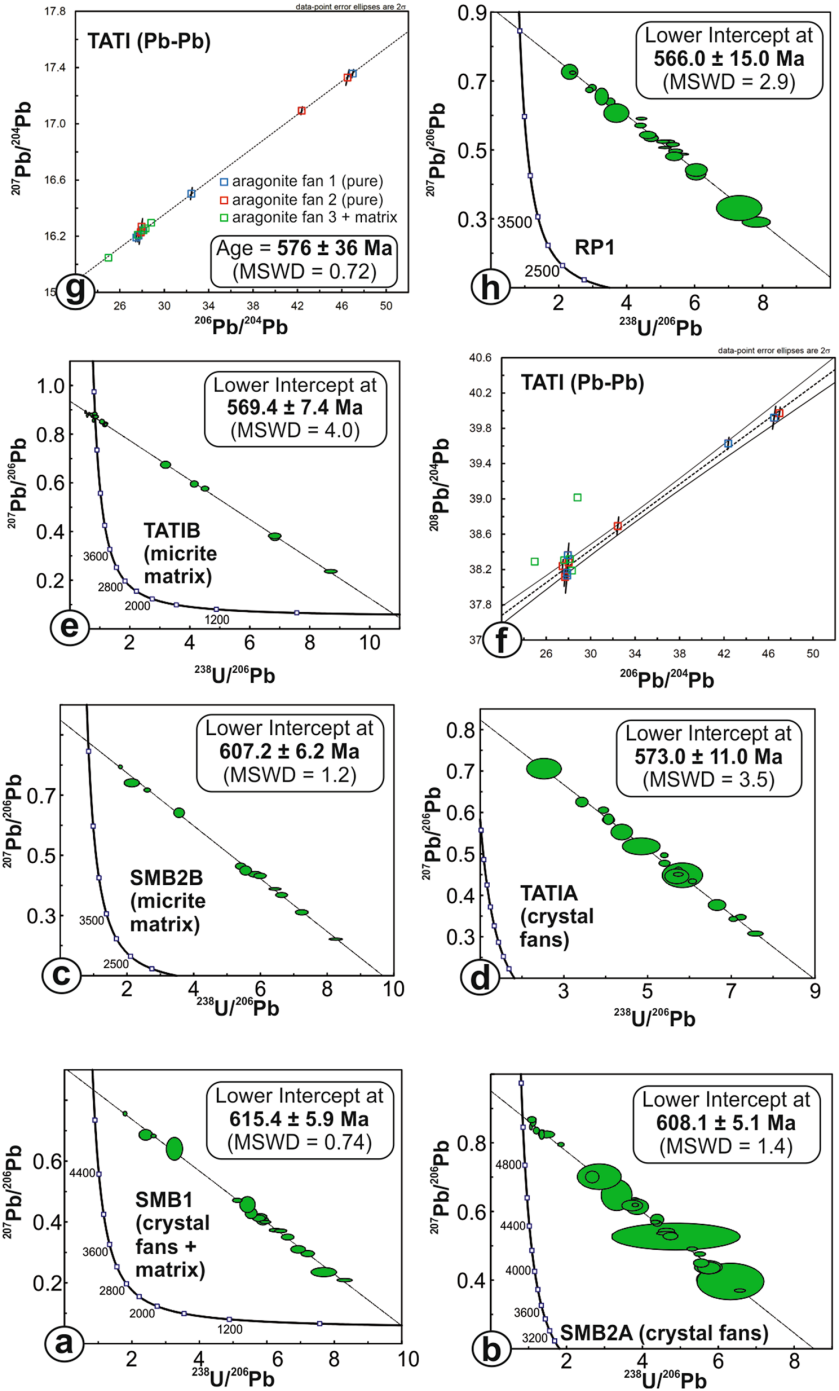
U–Pb and Pb-Pb plots of the analyzed samples. Charts were created using the Isoplot 3.6 Visual Basic Add-in freeware by Ken Ludwig, Berkeley Geochronology Center (http://www.bgc.org/isoplot) for Microsoft Excel (http://www.microsoft.com).

Two samples from crystal-fan-bearing limestone of the Pedro Leopoldo Member at the Sambra Quarry (Fig. 2a) with negative δ^13^C_carb_ typical of cap carbonate units (Fig. 2b) yielded U–Pb lower intercept dates of 615.4 ± 5.9 Ma (SMB1 − crystal-fans + matrix), 608.1 ± 5.1 Ma (SMB2A − crystal-fans) and 607.2 ± 6.2 Ma (SMB2B − matrix) (Fig. 3a–c) and a mean in-situ ^87^Sr/^86^Sr ratio of 0.707224 ± 0.000006 (Fig. 2c).

Above the LBH, the basal crystal-fan-bearing limestone of the Lagoa Santa Member at the Tatiana Quarry (Fig. 2d) yielded lower intercept U–Pb dates of 573.0 ± 11 Ma (TATIA − crystal-fans; ^87^Sr/^86^Sr of 0.707525 ± 0.000007) and 569.4 ± 7.4 Ma (TATIB − matrix; ^87^Sr/^86^Sr of 0.708058 ± 0.000017) (Fig. 3d,e). The same samples yielded a 576 ± 36 Ma stepwise Pb leaching isochron (Fig. 3f, g). New carbon isotope data present homogeneous δ^13^C around 0‰ and ^87^Sr/^86^Sr whole-rock data for the entire section are between 0.7078 and 0.7083 (Fig. 2e,f).

The topmost dark stromatolitic carbonates of the Lagoa Santa Member (Fig. 2g) at the Road Police Station near Sete Lagoas bear very positive δ^13^C > 10‰ corresponding to the MIBE (Fig. 2h) and yielded a 566 ± 15 Ma U–Pb date (Fig. 3h), with in-situ ^87^Sr/^86^Sr = 0.707270 ± 0.000012 for the stromatolite columns and 708,140 ± 0.000029 for the dark micrite matrix (Fig. 2i).

**Figure 4:**
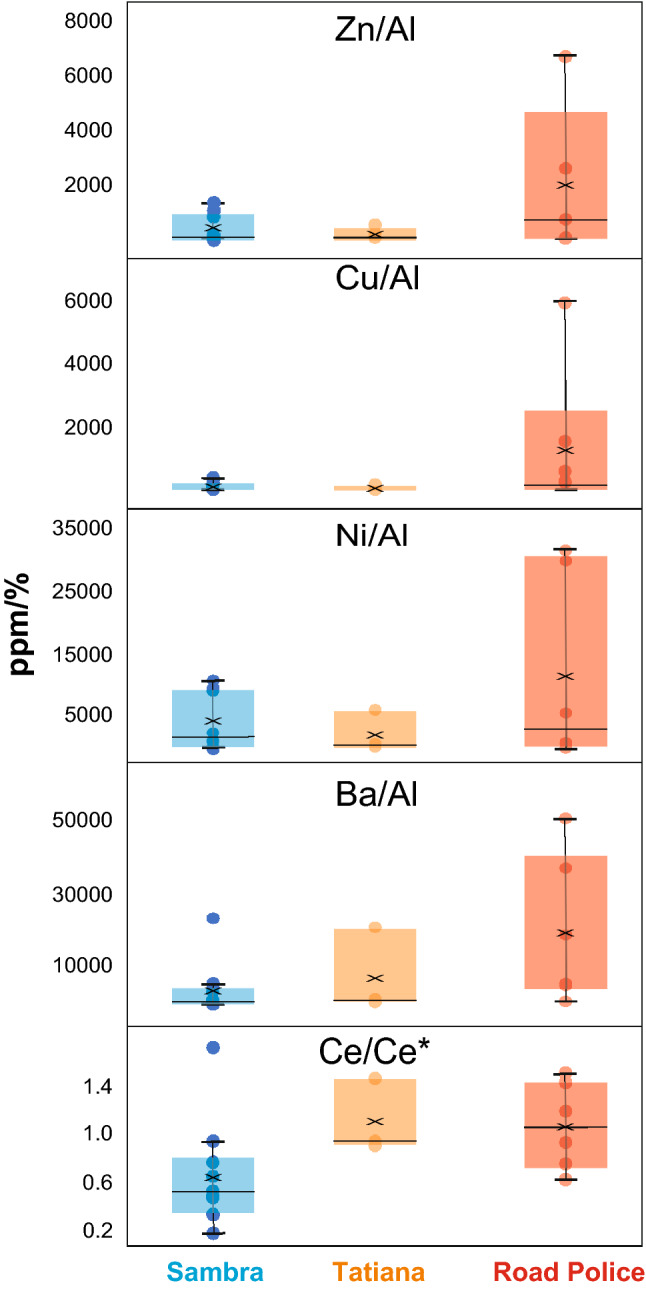
Box–Whisker plots showing the variation of trace metal contents and Ce anomalies for each studied section. Charts were created using Microsoft Excel (http://www.microsoft.com) and Corel Draw Graphics Suite 2018 (http://www.coreldraw.com).

In-situ trace element data indicates wide variations between the three studied sections. Box–Whisker plots (Fig. 4) show that there is a circa five-fold increase in Al-normalized trace metals such as Zn, Ni, Cu, and Ba in the Road Police section compared to the Sambra and Tatiana sections. The PAAS-normalized REE + Y patterns are LREE-depleted or MREE-enriched, with Y/Ho ratios between 35 and 61 and no Eu anomalies. The main distinctive traits between the sections are the Ce anomalies, which are prominently negative in the Sambra section (down to 0.2) and variable to null in the Tatiana and Road Police sections.

## Discussion

**Figure 5:**
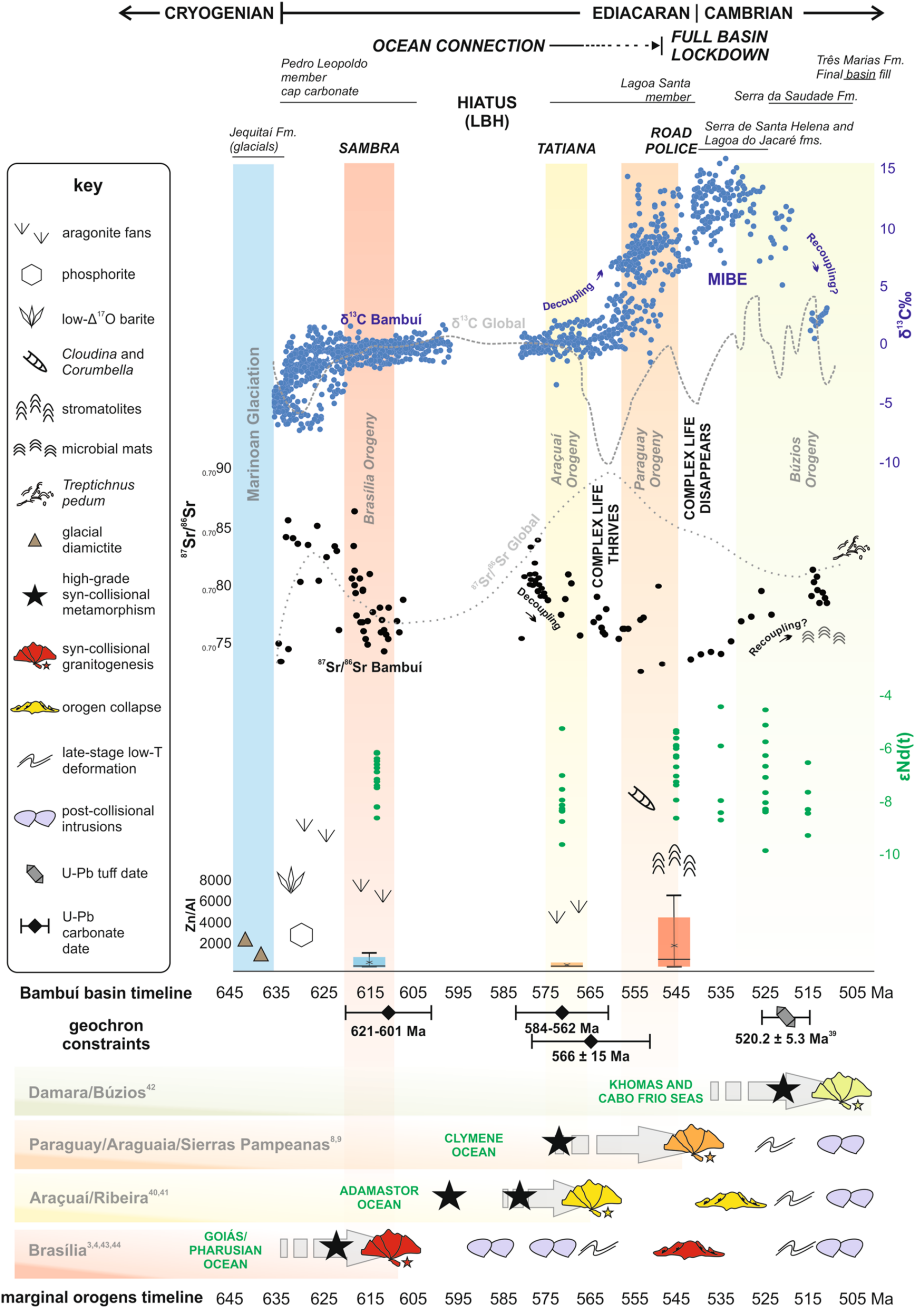
Timeline showing the integrated evolution of mountain belts and metazoan-bearing basins at the core of western Gondwana. The new data provided here is combined in a comprehensive compilation of C (blue dots) and Sr (black dots) isotope data from the literature, interpreted in the framework proposed here and compared to the global carbon and strontium isotope curves^36^. Literature-compiled Nd isotope data (in green, with poorer stratigraphic control than C and Sr data) is also presented for comparison. The amount of nutrient input in each evolutionary stage of the basin is represented by Box–Whisker plots of Zn/Al ratios, color-coded according to Fig. 4. Figure created using Microsoft Excel Professional Plus 2016 (http://www.microsoft.com), Corel Draw Graphics Suite 2018 (http://www.coreldraw.com) and a Huion Kamvas GS1331B pen display (http://www.huion.com).

**Figure 6:**
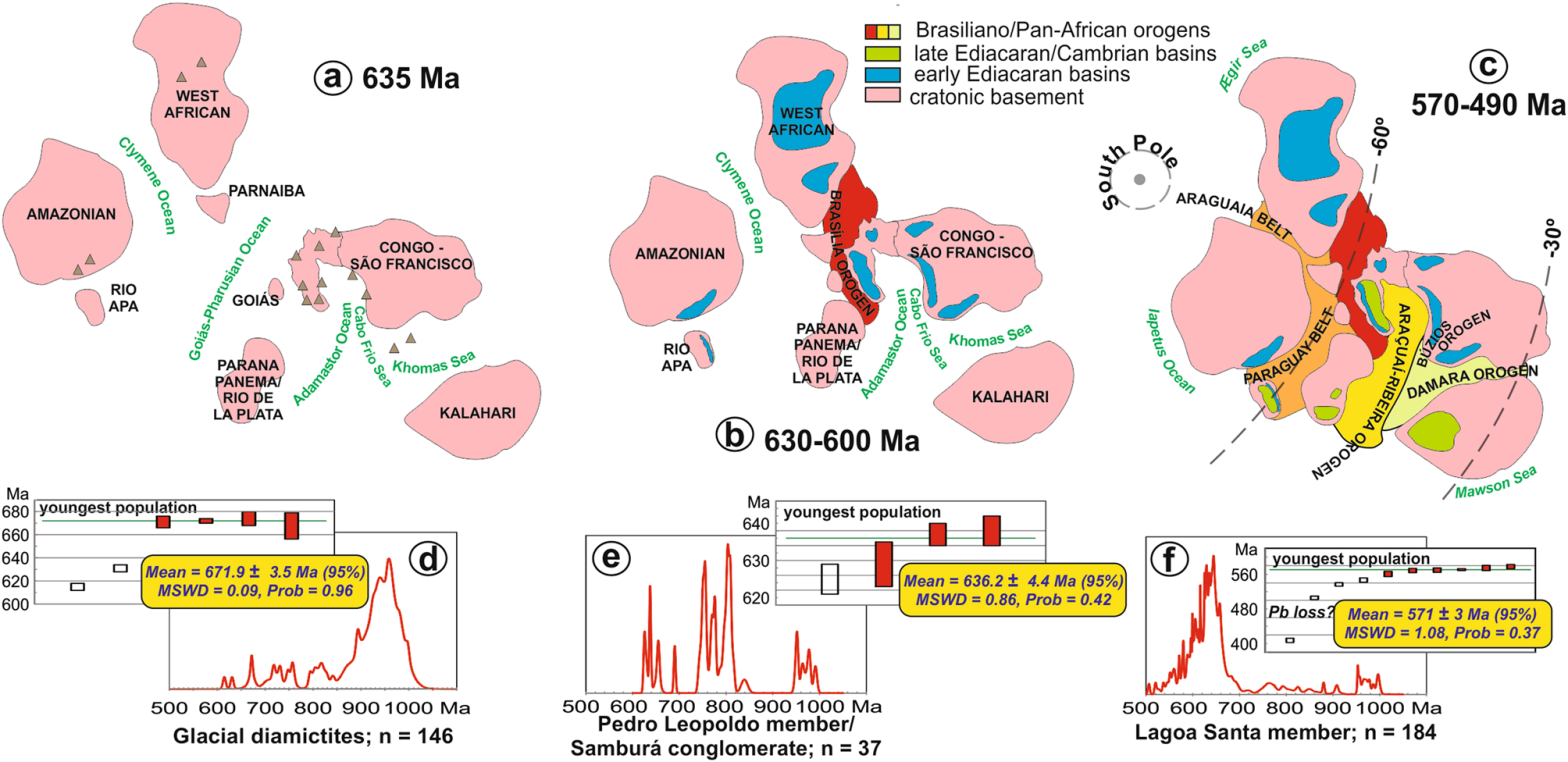
Integrated evolution of Ediacaran–Cambrian orogens and basins in western Gondwana. In (**a**–**c**) schematic models of evolution of western Gondwana during the late Ediacaran-early Cambrian. In (**d–f**) literature-compiled Neoproterozoic detrital zircon ^206^Pb/^238^U age spectra for the Bambuí basin during each of the stages depicted in (**a–c**) respectively. Approximated paleolatitudes in c from^37^. Charts were created using the Isoplot 3.6 Visual Basic Add-in freeware by Ken Ludwig, Berkeley Geochronology Center (http://www.bgc.org/isoplot) for Microsoft Excel (http://www.microsoft.com), and the maps were created using Corel Draw Graphics Suite 2018 (http://www.coreldraw.com) and a Huion Kamvas GS1331B pen display (http://www.huion.com).

Mountain building was diachronous in western Gondwana due to protracted collision of the São Francisco-Congo, West African, Paranapanema/Rio de La Plata, Pampean(?), Amazonian and Kalahari paleocontinents and the minor intervening continental blocks^18^ (Figs. 5, 6). Closure of the Goiás-Pharusian ocean generated the first major collisional belt, constrained at ca. 630–600 Ma from the Tuareg Shield in the Saharan region to the Brasília Orogen in central Brazil^11,18^. This orogen was built through collision of the West African and Paranapanema/Rio de La Plata paleocontinents with the northwestern and southwestern margins of the São Francisco paleocontinent, respectively (Figs. 5, 6a,b).

After ca. 15 Myr, syn-collisional crustal anatexis and peak metamorphic conditions were attained in the Araçuaí-Ribeira Orogen to the east of the São Francisco paleocontinent, marked by widespread 585–530 Ma aluminous granites and high-grade rocks^19^. This orogen was formed through closure of the V-shaped Adamastor ocean^12^, with collision of the Paranapanema/Rio de La Plata, São Francisco-Congo and Kalahari paleocontinents (Figs. 5, 6c). The Khomas and Cabo Frio seaways, as remnant arms of the Adamastor ocean, were further closed during the Cambrian generating the Damara and Búzios orogens, respectively^18^.

Finally, collision of the Amazonian paleocontinent and closure of the Clymene ocean ensued at 540–500 Ma, generating the Araguaia-Paraguay-Pampean Orogen and concluding the final amalgamation of western Gondwana during the Jiangshanian Series of the middle Cambrian^20^ (Figs. 5, 6c). The far-field stresses generated by this collision reactivated the external structures of the Brasília Orogen in an intracontinental context, as marked by Ar–Ar dates and youngest detrital zircons of ca. 540 Ma in the frontal nappes that thrust the Bambuí basin to the west^18,22^.

In Fig. 5, the new geochronological, isotopic and trace element data presented here is integrated in a comprehensive compilation of C, Sr and Nd isotope data from the available literature. Interpreted together in the framework proposed here, the amassed dataset establishes a chrono-correlation of the Bambuí Group evolutionary stages and diachronous mountain belt formation around the São Francisco paleocontinent. These can be summarized in a three-stage evolution:

(1) *Goiás-Pharusian ocean closure, Brasília Orogen building and Marinoan deglaciation (ca. 635–615 Ma)—*At the beginning of this stage, the São Francisco paleocontinent was surrounded by oceans (Fig. 6a). Closure of the Goiás-Pharusian ocean and building of the Brasília Orogen proceeded during this stage^11,18^. Carbon and strontium isotopes in the Pedro Leopoldo Member mirror the global early Ediacaran curve (Fig. 5), due to continued seawater connection through the Adamastor ocean (Fig. 6b). The negative Ce anomalies detected in the Sambra Quarry crystal-fan bearing carbonates (Fig. 4) are consistent with oxic conditions in the water column^38^, especially for high-purity carbonate samples with low detrital and organic matter content such as crystal-fan precipitates. This additional proxy supports the previous interpretations of oxic conditions for the surficial waters of the Bambuí basin in the aftermath of the Marinoan glaciation, based on Cr isotope^25^, Mo and U enrichments and Fe speciation^29^ data for this same interval. This, however, seems to have been a temporary and patchy oxygenation pulse, perhaps triggered by glacial meltwater input to the basin^25^. Nevertheless, it led to an important input of sulfate and phosphate to the basin, with formation of phosphorites^26^ and authigenic phosphate cements encrusting crystal fans^27^. Ca isotope systematics^39^, as well as the sharp ^87^Sr/^86^Sr peak from ca. 0.7074 to ca. 0.7080, support enhanced weathering of source areas during deposition of the cap dolostone.

The LBH is here constrained to between ca. 600 and 580 Ma, according to the U–Pb in-situ dates, within uncertainties, obtained in the Pedro Leopoldo and Lagoa Santa members, respectively (Fig. 3). This matches the time interval between closure of the Goiás-Pharusian and Adamastor oceans^11,12,18^ (Fig. 5) and roughly coincides with the onset of the regional Gaskiers glaciation, recognized in parts of South America^40^. Considering the U–Pb in-situ dates obtained here, along with the available field, lithostratigraphic and isotope data, the Pedro Leopoldo member would represent the cap carbonate to the Marinoan glaciation, deposited between 635 and 600 Ma and bearing consistent δ^13^C, ^87^Sr/^86^Sr and typical features such as barite layers with negative Δ^17^O and phosphorite deposits, while the Lagoa Santa member would represent carbonates deposited after a ca. 20 Ma depositional hiatus and spanning from ca. 580 to at least ca. 550 Ma, entering the *Cloudina sp.* biozone.

(2) *Adamastor ocean closure, Araçuaí Orogen building and optimum conditions for life development (ca. 585–530 Ma)—*Uplift of the Araçuaí-Ribeira Orogen at ca. 585–530 Ma caused renewed flexure of the São Francisco paleocontinent lithosphere, generating new accommodation space. At that time, the Bambuí basin was still connected to the Ediacaran global ocean, recording ^87^Sr/^86^Sr ratios of ca. 0.7080^41^ and δ^13^C_carb_ around 0‰^21,23^. Late-Ediacaran biomineralizing metazoans such as *Cloudina* sp.^15^ briefly thrived.

The disappearance of Ce anomalies in the Tatiana quarry limestones supports the available Fe speciation and trace metal proxies^29^ that depict a significant change from a likely pervasively oxic basin in the aftermath of the Marinoan glaciation (Sambra quarry) to unstable marine redox conditions. Although this situation might be interpreted as hazardous to life, previous studies revealed the ability of the first metazoans to colonize the marine substrate during sporadic oxic episodes under a regime of dominantly anoxic water conditions^10,42^, for example in the late Ediacaran Nama Group^42–44^.

The cause for the shift in redox conditions between the Pedro Leopoldo and Lagoa Santa members is uncertain. At ca. 585–530 Ma the Bambuí basin was progressively surrounded by orogens, and erosion of the uplifting mountain belts probably enhanced delivery of sediments and nutrients. High rates of primary productivity may cause a general drawdown of dissolved oxygen if the amassed biomass is subjected to aerobic remineralization and organic carbon is not buried fast enough. The proximity with several mountains probably caused local high sedimentation rates that resulted in increased rates of biomass burial, thus proportionally increasing inner-ramp dissolved O_2_ concentrations and enabling fleeting colonization of the cosmopolitan *Cloudina* genus during a time of heterogeneous redox conditions. The case of the Bambuí Group reinforces previous interpretations that delivery of an optimum amount of nutrients from oxidative weathering of mountains, combined with a balance between primary production and organic matter burial, were probably essential to opportunistic benthic colonization during times of ephemeral oxygenation among episodic incursion of anoxic waters beneath a fluctuating chemocline ^42,44–47^.

(3) *Clymene ocean closure, Paraguay-Araguaia Orogen building and full basin restriction during the MIBE interval (540–500 Ma)—*Collision of the Amazonian paleocontinent and consequent closure of the Clymene ocean to the west^20^ caused renewed uplift of the Brasília Orogen (Fig. 6c), completely restricting the Bambuí basin from all sides. These processes were ultimately responsible for the unique Middle Bambuí Excursion (MIBE) of δ^13^C_carb_ > 10‰^23^ (Fig. 5). The ^87^Sr/^86^Sr ratios became decoupled from the global curve, recording anomalously low values of circa 0.7072 (Fig. 5), probably due to the erosion of juvenile terranes and/or of ancient carbonate platforms uplifted in the surrounding orogenic belts^23,41^ (Fig. 6). This interpretation is reinforced by the available Nd isotope data compiled from the literature, which shows a slight increase of εNd(t) values during the MIBE, roughly coincident with the minima of ^87^Sr/^86^Sr values (Fig. 5). This rise in εNd(t) values is consistent with the suggestion of increased weathering of juvenile terranes in the orogenic belts that surrounded the Bambuí basin, potentially increase the delivery of key nutrients such as phosphorus from the erosion of basic and intermediate rocks^48^. This is reinforced by the presence of detrital apatite in carbonates of the MIBE interval^32^.

This possible large increase in nutrient input is consistent with the five-fold rise in the concentration of micronutrients such as Zn, Cu, Ni and Ba in the Road Police section, upper Lagoa Santa Member (Fig. 4). Metal enrichment was probably controlled by both weathering flux, as evidenced by paired low ^87^Sr/^86^Sr and high εNd(t), and by anoxic conditions stablished for this stratigraphic interval from Fe speciation and trace element concentrations^29^. In addition, detrital apatite found in carbonates of the MIBE interval^32^ could indicate, besides sourcing from basic to intermediate rocks, strong recycling of the PO_4_-rich basal carbonate platforms and re-fertilization of the basin. In this scenario, progressive restriction due to tectonic confinement and uncontrolled delivery of nutrient-rich waters might have fueled biomass production that was efficiently re-mineralized through methanogenesis^41,49^. Limestones that record the MIBE present a covariation of paired δ^13^C_carb_- δ^13^C_org_ data, high δ^34^S_pyrite_ and low carbonate-associated sulfate (CAS)^31,32^. A sulfate poor, water-column methanogenesis environment was recently proposed^31,32^, in which ^13^C-depleted methane is released from sediments and water without being oxidized. Thus, seawater DIC record anomalously positive δ^13^C_carb_ signals from methanogenic ^13^C-enriched CO_2_^31,32^. This scenario is only achieved after a drastic reduction of the dissolved oxygen pool through aerobic respiration and consumption of other oxidants.

Methanogenesis in the water column, instead of porewater methanogenesis, is supported by the basinal scale of the MIBE, occurring for hundreds of meters in distinct portions of the basin with little sample-to-sample variation, including in oolitic limestones deposited under shallow and agitated waters^32^. Alternative factors might have contributed to the MIBE, such as a change in total carbon input from the weathering of older carbonate rocks with high δ^13^C_carb_^23,41^ and a third authigenic carbonate sink^32^, but are unlikely to have acted alone in maintaining a ^13^C-enriched DIC throughout the basin^32^. A giant Ediacaran graphite deposit interleaved in paragneisses of the Araçuaí Orogen^50^ was proposed to potentially represent at least part of the organic carbon buried to generate the MIBE^32^, but there is a seeming mismatch in the constrained ages of the two events, as the graphite deposit was metamorphosed to high-grade at ca. 585–560 Ma^50^ and the MIBE would only have started after the beginning of the *Cloudina* sp. biozone, i.e. after ca. 550 Ma^2^.

While progressive basin restriction disconnected the Bambuí waters from the global sea and thus isolated it from the prevailing biotic and abiotic conditions, preventing the rise of typical late Ediacaran/early Cambrian macrofaunal biota, some distinct microbial benthic assemblages might still have thrived, as indicated by localized stromatolites, recently described MISS structures^23^ and putative ichnofossils such as *Treptichnus pedum* in the upper Bambuí Group^51^. It should be noted, however, that microbial mats of the Jaíba Formation, situated below the unit from which the possible ichnofossils were described (Três Marias Formation), yielded δ^13^C_carb_ around + 3 ‰ and ^87^Sr/^86^Sr of ca. 0.7080^52^, similar to the early Cambrian seawater curve, which might suggest a recoupling with global oceanic waters and the return to normal biogeochemical conditions at the topmost Bambuí Group after the intensively restricted period of the MIBE (Fig. 5).

Inner-ramp stromatolites of the Road Police section yielded Ce anomalies that are consistent with the interpretation of anoxic seawater from other proxies such as Fe speciation and U and Mo contents for the same stratigraphic interval^29^. A significant shallowing of the redoxcline and increasing of anoxic incursions into proximal settings during the MIBE is thus interpreted. Anaerobic degradation of organic matter (through methanogenesis and dissimilatory Fe reduction) likely resulted in a degree of P recycling back to the water column, thus fueling productivity to probably very high levels^53^, provided P did not get trapped in ferrous iron minerals.

The absence of oxygen and a probable methane-rich water made the environment extremely eco-stressful and hampered complex life forms and the establishment of a typical Ediacaran–Cambrian style trophic chain^29^. We suggest that during the MIBE an overburden of nutrients was prejudicial for metazoan diversification, such as recently proposed for other Late Ediacaran and Phanerozoic basins^44,54^ and constrained from numerical models^55^. This tentative model needs to be further tested and confirmed by a systematic study using other nutrient proxies in different parts of the basin. Nevertheless, available coupled δ^13^C_carb_-δ^13^C_org_ data^31,32^, high δ^34^S_pyrite_ and low CAS^32^ throughout the MIBE indicate the attainment of primary production under low-sulfate and low-oxygen conditions, reinforcing the suggestion of a dominantly ferruginous water column modeled through Fe speciation and trace element data^29^ for this time interval.

A compilation of published detrital zircon data (Fig. 6) supports the evolutionary model proposed here. Provenance of the Marinoan glacial diamictite and related units reflects the erosion of mainly cratonic sources, with the main Neoproterozoic detrital zircon peak at ca. 900 Ma and sparse crystals with a youngest peak at 670 Ma^56,57^ that might have been transported in volcanic ash clouds derived from surrounding island arcs^12^ (Fig. 6a,d). Building of the Brasília Orogen^18^ provided youngest detrital zircons of ca. 636 Ma to foredeep conglomerate wedges that developed roughly during the time of cap carbonate deposition^22^ (Fig. 6b,e). An important provenance shift is observed within the fossil-bearing limestone, with youngest detrital zircons at ca. 570 Ma^58^, indicating erosion of the Araçuaí mountain belt (Fig. 6c, f). The 520.2 ± 5.3 Ma^33^ U–Pb zircon date interpreted as the age of extrusion of a tuff layer at the top of the Bambuí Group indicates that deposition spanned the time of closure of the Clymene ocean and final amalgamation of western Gondwana.

The relationship between orogens and metazoan-bearing basins proposed for Ediacaran/Cambrian systems^7,8,11^ is thus more complex than previously thought. According to our model, mountains might provide the conditions for life development, i.e., delivery of bio-essential nutrients, causing a boost in primary productivity and the subsequent rise of atmospheric and ocean oxygen levels. However, mountains may hinder complex life development if basins become too restricted by the surrounding uplifted areas that hamper seawater renewal and encourage eutrophication from an excess of nutrients and biomass production. There is, then, a Goldilocks effect constraining the optimum conditions for metazoan development, especially in Ediacaran–Cambrian basins surrounded by mountain belts formed due to Gondwana assembly. This effect might be recognizable in other moments of geological history as well^54^.

The effect of extreme restriction of Ediacaran–Cambrian sedimentary basins is not, however, restricted to local conditions for life development. Most Neoproterozoic-Cambrian rift to passive-margin sedimentary basins of Laurentia, Baltica and Siberia, which during the Neoproterozoic were detached from Rodinia, remained likely open to the global sea. In contrast, various intracontinental sedimentary basins now preserved in the southern hemisphere continents became progressively restricted and landlocked towards the end of the Ediacaran, with fan-like approximation and collision of Rodinia’s offspring as the building blocks of Gondwana^59^. Wide methanogenic, anoxic and low-sulfate basins in the interior of Gondwana might have caused an important input of large quantities of methane to the atmosphere, as recently suggested^31,32^, thus potentially affecting the global carbon cycle and climate. In effect, recent studies suggest that the early Cambrian global climate was characterized by greenhouse conditions, similar to the late Mesozoic and early Cenozoic greenhouse climates, based on δ^18^O signatures preserved in fossil biogenic phosphate^60^. Thus, a very important future research direction is to quantify the degree of restriction and the influence of Gondwanan basins, preserved in present-day southern hemisphere continents, in Earth’s global biogeochemical cycles.

## Methods

### Carbon, oxygen and strontium isotope ratio mass spectrometry

Carbonates had their CO_2_ extracted on a high vacuum line after reaction with phosphoric acid at 25 °C, and cryogenically cleaned at the Stable Isotope Laboratory (NEG-LABISE) of the Department of Geology, Federal University of Pernambuco (UFPE), Brazil. Released CO_2_ gas was analyzed for O and C isotopes in a double inlet, triple collector mass spectrometer (VG-Isotech SIRA II), using the BSC reference (Borborema Skarn Calcite) that was calibrated against NBS-20 (δ^13^C = − 1.05‰_VPDB_; δ^18^O = − 4.22‰_VPDB_). The external precision, based on multiple standard measurements of NBS-19, was better than 0.1‰ for both elements.

Aliquots of the carbonate samples were attacked with 0.5 M acetic acid in order to prevent dissolution of the siliciclastic fraction, following procedures described in^21^. Sr was then separated using the conventional cation exchange procedure at the Laboratory of Geochronology, University of Brasília (UnB), Brazil. Samples were measured at 1250–1300 °C in dynamic multi-collection mode in a Thermoscientific Triton Plus mass spectrometer. The ^87^Sr/^86^Sr values of the samples were corrected for the offset relative to the certified NIST SRM 987 value of 0.710250. The long-term (year-round) average of this standard ^87^Sr/^86^Sr ratios measured in this machine is 0.71028 ± 0.00004. Procedural blanks for Sr are less than 100 pg. All uncertainties are presented at the 2σ level.

### U–Pb laser ablation ICP-MS dating

U–Pb dating of calcite via laser ablation-inductively coupled plasma-mass spectrometry (LA-ICP-MS) at the Applied Isotope Research labs of the Federal University of Ouro Preto (UFOP, Brazil) used a modified methodology by^61^. The ages were acquired in polished slabs from five hand-specimens in a 193 nm ArF excimer laser (PhotonMachines) equipped with a Hellex two volume ablation cell. The laser was coupled to a ThermoScientific Neptune Plus MC-ICP-MS. Samples were ablated in a helium atmosphere (0.2 L min^−1^) and mixed in the gas line with 0.99 L min^−1^ argon and 0.03 L min^−1^ nitrogen. Signal strength at the ICP-MS was tuned for maximum sensitivity while keeping oxide formation (monitored as ^248^ThO/^232^Th) below 0.2% and no fractionation of the Th/U ratio. Static ablation used a spot size of 80 µm and a fluence of about 1 J cm^−2^ at 8 Hz. Data were acquired in fully automated mode overnight in sequences of 150 to 260 analyses. Each analysis consisting of 20 s background acquisition followed by 35 s of sample ablation and 15 s washout. During data acquisition, the signals of ^206^Pb, ^207^Pb, ^208^Pb, ^232^Th and ^238^U were measured simultaneously in a collector block equipped with 9 faradays and 5 ion counters. Prior to analysis, each spot was pre-ablated 30 for 3 s to remove surface contamination. Soda-lime glass NIST SRM-614 was used as a reference glass together with one carbonate reference material (WC-1) and three other carbonate reference materials (see below). Raw data were corrected online using our in-house Saturn software, developed by João Paulo Alves da Silva. Following background correction, outliers (± 2σ) were rejected based on the time-resolved ^207^Pb/^206^Pb and ^206^Pb/^238^U ratios. The mean ^207^Pb/^206^Pb ratio of each analysis was corrected for mass bias (0.3%) and the ^206^Pb/^238^U ratio for interelement fractionation (~ 5%), including drift over the sequence time, using NIST SRM-614. Effects for mass bias and drift correction on the Pb/Pb ratios were monitored using USGS BCR and BHVO glasses. Over the three-day analyses compiled Pb/Pb ratios for both standards were within error to certified values by^62^.

Due to the presence of carbonate matrix, an additional offset factor of 1.3 was determined using WC-1 carbonate reference material^63^. The ^206^Pb/^238^U fractionation during 20 s depth profiling was estimated to be 3%, based on the common Pb corrected WC-1 analyses, and has been applied as an external correction to all carbonate analyses. Pooled together the U–Pb data for WC-1 obtained during the sections yielded an age of 254.5 ± 1.6 Ma (MSWD = 0.78, n = 53). Repeated analyses of a stromatolitic limestone from the Cambrian-Precambrian boundary in southern Namibia, analyzed during the sequences yielded a lower intercept age of 548 ± 16 Ma (MSWD = 3, n = 15). This is within uncertainty identical to the U/Pb zircon age of 543 ± 1 Ma from the directly overlying ash layer (Spitskopf Formation^64^). Repeated analyses of the Duff Brown carbonate yielded a lower intercept age of 63.85 ± 0.77 Ma (MSWD = 1.5, n = 17), which is within error of the age of 64.0 ± 0.7 Ma^65^. Lastly, fifteen spots on our internal reference material Rio Maior calcite gave a low intercept age of 62.43 ± 0.37 Ma (MSWD = 0.7, n = 15). This age is identical to our long-term measurement in the Department of Geology at Federal University of Ouro Preto (UFOP).

### In-situ LA-ICPMS Sr isotope ratios determination

A ThermoFisher Neptune Plus LA-(MC)-ICP-MS coupled with a 193 nm HelEX Photon Machine laser ablation system was used to obtain the Sr isotope composition of the carbonate samples at the Applied Isotope Research labs, Department of Geology, UFOP, Brazil. The methodology followed was proposed by^66,67^. Analytical conditions included a 6 Hz repetition rate and an energy density of 4 J cm^−2^ with a spot size of 85 µm. The acquisition cycle consisted of 30 s of measurement of the gas blank, followed by 60 s of sample ablation. Ablation and material transport occurred in a sample gas stream of Ar (0.8 l min^−1^) mixed with He (0.5 l min^−1^) and N_2_ (0.005 l min^−1^). The dataset was reduced using an in-house Excel® spreadsheet for offline data reduction (modified from^66^). The raw data for ^86^Sr/^87^Sr were corrected for the 84 and 86 isobaric mass interference (^84^Kr and ^86^Kr on ^84^Sr and ^86^Sr), using a Kr baseline measurement. Corrections for Er, Yb and Ca dimers had only a negligible effect during all sessions. At the beginning of each analytical session, soda-lime glass SRM-NIST 610 was measured 2–3 times for empirical determination of ^87^Rb/^85^Rb mass bias using the Sr mass bias (^86^Sr/^88^Sr relative to ^86^Sr/^88^Sr true = 0**.**1194). All samples followed measurements of BHVO glass, MIR (in-house plagioclase reference material from Dr. A. Gerdes, Frankfurt), Madagascar apatite reference materials, for quality control purposes. Over the analyses, MIR yielded values of ^87^Sr/^86^Sr = 0.70306 ± 1 (2σ, n = 30), in agreement (within uncertainty) with conventional TIMS data of 0.70309 ± 7 (2σ)^68^. For BHVO-1 ^87^Sr/^86^Sr = 0.70343 ± 14 (2σ, n = 3) are in agreement with a ^87^Sr/^86^Sr reference of 0.703436 ± 2 (2σ)^69^. ^87^Sr/^86^Sr ratios of Madagascar apatite (^87^Sr/^86^Sr = 0.711712 ± 22, 2σ, n = 6) were within the uncertainty of published mean of ^87^Sr/^86^Sr 0.71179 ± 3 (2σ)^70^ by LA-MC-ICP-MS. Finally, we have obtained ^87^Sr/^86^Sr ratios of a modern coral sample (^87^Sr/^86^Sr = 0.70915 ± 7 (2σ), n = 18) that is within uncertainty of published values of the modern seawater (^87^Sr/^86^Sr = 0.70917 ± 3 (2σ)) according to^71^.

### In-situ LA-ICPMS trace element analysis

Trace element composition of the calcite samples were obtained via LA-ICP-MS (CETAC 213 laser ablation coupled to a ThermoFisher Element 2) at the Applied Isotope Research laboratories, Department of Geology, UFOP, Brazil. The laser was set to produce spot sizes of 40 μm in diameter, during a period of 30 s at 10 Hz frequency. The data acquisition was done in bracketing mode and consisted of 4 analyses of standards (NIST 612 50 ppm glass) bracketing 10–15 unknowns. The data reduction was done via the Glitter software (GEMOC Laser ICP MS Total Trace Element Reduction), which provides an interactive environment for analytic selection of background and sample signals^72^. Instrumental mass bias and ablation depth-dependent elemental fractionation were corrected by tying the time-resolved signal for the unknown to the identical integration window of the primary standard NIST612. BCR and BHVO were used as secondary control reference materials, and yielded values within the recommended USGS range. Errors are derived from the averaged counts for each mass for both the standards and values are then compared to those of the primary and secondary standards, to determine concentrations.

### Stepwise Pb leaching dating

#### Analytical details

Rock samples bearing crystal fans were carefully cut out from a 5 mm thick slab with a rotating diamond blade mounted to a hand-hold hand-piece used in dental offices. The material was carefully ground by hand in an agate mortar, washed in deionized water, dried in an oven at 50° and then sieved into a 10–40 µm fraction. We prepared three aliquots: Two fractions (A1, A2) which we processed further by subjecting them to magnetic separation using a Frantz Isodynamic separator, cleaning the sample material from magnetic particles in the rock matrix and also intergrown directly with aragonite; and a third fraction (A3) which were not treated nor purified.

We performed stepwise Pb leaching^73^ (TATI sample) on all three sample aliquots (200 mg sample amounts) using 2 mL of different acids (HBr, acetic acid, HCl and aqua regia) with concentrations specified in Supplementary Table S3 and reaction times of 10 min in every step. Supernatants in every step were centrifuged, pipetted off and then transferred to clean 7 mL Savillex™ Teflon beakers. A fifth of each sequential liquid aliquot of samples A1 and A3 was pipetted into separate beakers and an adequate volume of a ^204^Pb enriched tracer solution was added to them. This allowed the precise determination of Pb amounts released during each and every sequential leaching step. Respective Pb concentrations, referred to the original 200 mg of material used at the beginning, are also contained in the Supplementary Table S3.

All sequentially leached samples were dried and, after conversion to the chloride form with 1 mL of 6 N HCl, Pb was separated on miniaturized 1 mL pipette tip columns with a fitted frit, charged with 300 µL of Biorad™ AG1-X8 100–200 mesh anion resin, using a conventional HCI-HBr anion exchange procedure with doubly distilled acids diluted to our needs with ultrapure water provided by a Milli-Q® Reference Water Purification System, contributing a blank of less than 100 pg.

Pb was loaded together with 2 µL silica gel and 1 µL 1 M phosphoric acid and measured from 20 µm Re filaments on a 8 collector VG Sector IT mass-spectrometer in static mode. Mass fractionation amounted to 0.068 ± 0.011%/AMU (2σ, n = 85), determined on repetitive analyses of the NBS 981 Pb standard. Errors (reported at the 2σ level) and error correlations (r) were calculated after^74^. Isochron ages were derived using Isoplot 3.6^74^. Errors assigned to the isochrons are 2σ given in the 95% confidence interval.

#### Results/discussion

TATI data, color-coded with respect to the three different aliquots processed, are plotted in a Pb isotope ratio diagram of Fig. 3g. It is apparent from the data in Supplementary Table S3 that the acetic acids steps removed most Pb (~ 70%) from the crystal fan separates, in line with our expectation that this acid is capable of preferentially attacking and dissolving the carbonate. In all experiments, the subsequent stronger leaching acids (HCl, HNO_3_-HCl mixture and *aqua regia*) released Pb fractions with a significantly more radiogenic Pb isotope signature (Supplementary Table S3). While this might indicate Pb release from at least another subordinate phase with elevated U and Th relative to Pb, information deduced from the uranogenic–thorogenic common Pb diagram seems to indicate that this is unlikely. Instead, the leaching patterns in this diagram reveals a linear relationship of the data, with the exception of aliquot 3 (not purified by magnetic separation). A linear arrangement of TATI data in this diagram strongly supports the leaching of only one phase, in this case calcite pseudomorphs after aragonite, contributing Pb to the leaching acids. Conversely, the scattered leaching pattern of A3 in the uranogenic–thorogenic common Pb diagram reflects the presence of a multicomponent system with Pb contributed from phases likely having different U/Th.

Based on the above, the well-defined correlation line defined by TATI data of the pure crystal fan separates (A1 and A2) in the uranogenic common Pb diagram is interpreted as a true mono-mineral isochron, with a slope corresponding to an age of 576 ± 36 Ma (MSWD = 0.72). We interpret this age to indicate the timing of growth of the respective aragonite fans. The results strongly reveal the importance of removal of matrix phases, in this case from the crystal fans, to prevent erroneous interpretation of linear arrays in uranogenic Pb isotope diagrams as isochrons, whereas they instead signify mixing lines with no interpretable geological meaning.

### Carbon, strontium and neodymium data compilation

The δ^13^C (1597 samples), ^87^Sr/^86^Sr (103 samples) and Nd isotope data (66 samples) compiled in Fig. 5 come from the following sources:^21–23,25,35,41,75–82^. As distinct stratigraphic sections show different thicknesses, stratigraphic positioning of the data points was normalized to a common thickness for each formation. Nd isotope data are not reported with stratigraphic height tie-points, with exception of the data reported by^25^; thus, the εNd(t) values, recalculated for the expected age of deposition, were grouped in a single column for each unit. Only the less radiogenic ^87^Sr/^86^Sr results reported for each section in the cited literature, corresponding to samples with higher Sr concentration and lower Mn/Sr ratios, were used in the compilation.

### Detrital zircon compilation

For the compiled detrital zircon ^206^Pb/^238^U age probability density plots of Fig. 6d–f, data from the following works were recalculated and only the spots showing less than 5% discordance, low common Pb and low uncertainty were used. For our purposes, only spots with ^206^Pb/^238^U ages younger than 1000 Ma were compiled. Age of the youngest population is calculated as the weighted average ^206^Pb/^238^U age using the minimum three youngest age-equivalent spots of each dataset (red bars in Fig. 6d–f), with uncertainties presented at the 95% level. This method is known as *weighted average of the youngest three grains* or Y3Z^83^, generally considered successful and accurate for low-n datasets (n < 300). Spots with younger ages, but not age-equivalent to at least other two spots, are considered outliers, either reflecting Pb loss, low sample size, analytical issues or under-representation due to statistical or analytical bias, and are not considered as reliable indicators of maximum depositional age.

Glacial diamictites (Fig. 6d) include the cratonic Jequitaí and Bebedouro formations and correlated diamictite-bearing units in the marginal fold belts: the Canabravinha Formation of the Rio Preto belt, the Cubatão Formation of the Brasília belt, the lower diamictite-bearing formations of the Macaúbas Group of the Araçuaí belt and the Capitão-Palestina Formation of the Sergipano belt. The latter is capped by the Olhos D’água cap carbonate bearing identical C, O and Sr isotopic signals to the Pedro Leopoldo cap carbonate^21^. The sources for the compilation of Fig. 6d are:^56,57,77,84–88^. Figure 6e is plotted with detrital zircon data from the Samburá conglomerate wedge^22^, which is overlain by the Pedro Leopoldo cap carbonate on the western part of the basin. No Neoproterozoic detrital zircon data is available for the cap carbonate unit itself. Figure 6f presents detrital zircon data for the Lagoa Santa Member compiled from^58,77,78^.

## Supplementary Information

Supplementary Tables.

## Data Availability

All data are available within the paper and its Supplementary Material tables or from the corresponding author upon reasonable request.
