# Supplementary material for: Goldilocks at the dawn of complex life: mountains might have damaged Ediacaran–Cambrian ecosystems and prompted an early Cambrian greenhouse world
Source: Sci Rep. 2021 Oct 8;11:20010. doi: 10.1038/s41598-021-99526-z (PMC8501109; doi:10.1038/s41598-021-99526-z)
Supplement: Supplementary file 1 — Supplementary Tables. [file 41598_2021_99526_MOESM1_ESM.pdf]

Supplementary Files for "Goldilocks at the dawn of complex life: Mountains might have damaged Ediacaran-Cambrian ecosystems and prompted an early Cambrian greenhouse world"

Fabricio Caxito[1*], Cristiano Lana[2], Robert Frei[3], Gabriel J. Uhlein[1], Alcides N. Sial[4], Elton L. Dantas[5], André G. Pinto[1], Filippe C. Campos[1], Paulo Galvão[1], Lucas V. Warren[6], Juliana Okubo[6], Carlos E. Ganade[7]

1 CPMTC Research Center, Universidade Federal de Minas Gerais, Belo Horizonte, MG 31270-901, Brazil

2 Departamento de Geologia, Universidade Federal de Ouro Preto, Ouro Preto, MG 35400-000, Brazil

3 Department of Geoscience and Natural Resource Management, University of Copenhagen, Øster Voldgade 10, 1350 Copenhagen, Denmark

4 NEG-LABISE, Universidade Federal de Pernambuco, Recife, PE 50740-530, Brazil

5 Laboratório de Estudos Geodinâmicos, Geocronológicos e Ambientais, Universidade de Brasília, Brasília, DF 70910-900, Brazil

6 Departament of Geology, São Paulo State University, Rio Claro, SP 13506-900, Brazil

7 Geological Survey of Brazil – CPRM, Rio de Janeiro, RJ 22290-255, Brazil

* Corresponding author: Fabrício Caxito at caxito@ufmg.br

Table S1 - U-Pb LA-IPC-MS data for carbonate rocks from Sambra and Tatiana quarries and Road Police station and of reference carbonate material for U/Pb matrix offset correction and for quality control purposes

SAMBRA QUARRY - SMB1 (crystal-fans + micrite matrix) - Pedro Leopoldo cap carbonate member

SPOT	U(ppm)	Th/U	207Pb(cps)	206Pb(cps)	208Pb(cps)	207Pb/206Pb	2sigma(%)	207Pb/235U	2sigma(%)	206Pb/238U	2sigma(%)	Rho	206Pb/238U	2sigma(s)	207Pb/235U	2sigma(s)	207Pb/206Pb	2sigma(s)
SMB1 - 1	1.95	0.064	115357.84	172217.26	166986.23	0.685	2.1	39.112	6.9	0.414	6.5	1.0	2233	124	3748	70	4700	30
SMB1 - 2	1.05	0.021	53652.08	84039.32	78829.61	0.682	0.7	35.500	2.5	0.377	2.4	1.0	2064	43	3653	25	4694	10
SMB1 - 3	0.72	0.003	64877.92	85587.04	84606.72	0.755	0.8	57.756	2.6	0.555	2.5	1.0	2844	57	4136	26	4840	11
SMB1 - 4	2.53	-0.010	36509.23	91656.64	90477.02	0.403	2.8	9.440	3.7	0.170	2.5	0.7	1011	23	2382	35	3921	42
SMB1 - 5	2.21	0.006	92544.21	143965.35	142858.25	0.639	4.8	26.917	7.7	0.305	6.0	0.8	1718	91	3380	78	4600	69
SMB1 - 6	2.08	-0.009	31219.18	75796.03	74699.63	0.418	1.4	9.849	2.5	0.171	2.2	0.8	1018	20	2421	24	3974	20
SMB1 - 7	5.04	-0.004	32895.97	139455.46	139122.83	0.234	5.1	4.191	6.5	0.130	4.0	0.6	787	30	1672	55	3080	82
SMB1 - 8	7.35	-0.003	38070.26	188046.50	186334.81	0.207	2.1	3.428	3.1	0.120	2.2	0.7	731	15	1511	24	2882	34
SMB1 - 9	2.60	-0.011	39541.50	95027.52	94369.07	0.415	2.2	9.830	3.4	0.172	2.6	0.8	1022	25	2419	32	3965	32
SMB1 - 10	3.58	-0.006	31163.55	105611.53	105042.02	0.295	2.7	5.625	3.5	0.138	2.3	0.6	835	18	1920	31	3443	42
SMB1 - 11	3.38	-0.005	32118.00	103725.56	103250.48	0.309	3.3	6.130	4.1	0.144	2.5	0.6	868	21	1995	37	3514	50
SMB1 - 12	2.18	-0.002	27647.32	74053.18	73669.88	0.372	1.3	8.168	2.8	0.159	2.5	0.9	953	22	2250	26	3799	20
SMB1 - 13	2.71	-0.010	41428.08	99929.57	99502.62	0.411	2.1	9.809	3.4	0.173	2.6	0.8	1028	25	2417	31	3952	32
SMB1 - 14	2.61	-0.002	43086.44	100083.39	99653.89	0.427	3.3	10.606	4.2	0.180	2.6	0.6	1068	25	2489	40	4007	49
SMB1 - 15	2.10	-0.008	23440.76	67505.90	66928.12	0.349	2.2	7.269	3.2	0.151	2.3	0.7	906	20	2145	29	3705	33
SMB1 - 16	1.56	-0.015	13983.06	51645.50	44870.91	0.456	4.7	11.557	5.8	0.184	3.4	0.6	1087	34	2569	56	4106	70
SMB1 - 17	1.75	-0.014	25224.45	62607.00	62436.42	0.399	1.0	9.212	2.4	0.168	2.2	0.9	999	20	2359	22	3904	15
SMB1 - 18	1.79	-0.013	22228.12	59546.91	59359.14	0.370	1.4	7.939	2.7	0.156	2.3	0.9	933	20	2224	25	3791	21
SMB1 - 19	1.59	-0.010	31419.31	66061.46	65872.81	0.470	1.1	12.628	2.5	0.195	2.2	0.9	1147	23	2652	24	4150	16

SAMBRA QUARRY - SMB2A (crystal-fans) - Pedro Leopoldo cap carbonate member

SPOT	U(ppm)	Th/U	207Pb(cps)	206Pb(cps)	208Pb(cps)	207Pb/206Pb	2sigma(%)	207Pb/235U	2sigma(%)	206Pb/238U	2sigma(%)	Rho	206Pb/238U	2sigma(s)	207Pb/235U	2sigma(s)	207Pb/206Pb	2sigma(s)
SMB2A -1	2.20	0.004	37695.48	99288.95	37695.48	0.401	7.7	8.867	13.0	0.161	10.5	0.8	960	94	2324	126	3911	116
SMB2A -2	1.83	-0.003	32436.95	83688.65	32436.95	0.409	3.3	9.216	5.5	0.163	4.5	0.8	976	41	2360	52	3943	49
SMB2A -3	1.73	-0.007	29773.67	78408.91	29773.67	0.401	1.4	8.935	3.0	0.162	2.7	0.9	966	24	2331	28	3912	20
SMB2A -4	2.03	-0.007	33637.16	89939.24	33637.16	0.395	11.0	8.599	15.9	0.158	11.4	0.7	946	101	2296	156	3889	166
SMB2A -5	1.91	-0.002	28471.54	81502.91	28471.54	0.369	1.0	7.726	2.0	0.152	1.8	0.9	912	15	2200	19	3786	15
SMB2A -6	1.79	0.008	36072.15	87153.25	36072.15	0.437	3.6	10.481	6.3	0.174	5.2	0.8	1035	50	2478	61	4040	54
SMB2A -7	2.05	0.002	58027.51	116465.60	58027.51	0.525	6.0	14.843	28.9	0.205	28.3	1.0	1201	318	2805	321	4314	89
SMB2A -8	1.66	0.009	32198.23	77736.54	32198.23	0.437	1.0	10.214	2.3	0.169	2.1	0.9	1009	19	2454	21	4043	15
SMB2A -9	2.06	0.022	40771.12	99081.97	40771.12	0.434	3.2	10.397	5.4	0.174	4.3	0.8	1032	41	2471	51	4032	48
SMB2A -10	1.44	0.034	57786.71	100313.95	57786.71	0.608	1.2	21.140	3.1	0.252	2.9	0.9	1451	38	3145	31	4526	18
SMB2A -11	1.03	0.004	44303.01	74596.07	44303.01	0.627	1.6	22.688	3.6	0.263	3.3	0.9	1503	44	3214	36	4571	22
SMB2A -12	1.20	0.085	63214.97	101493.05	63214.97	0.657	1.4	27.627	4.7	0.305	4.5	1.0	1717	68	3406	48	4639	21
SMB2A -13	1.78	0.021	43017.43	92653.19	43017.43	0.490	1.0	12.718	2.3	0.188	2.1	0.9	1112	22	2659	22	4211	15
SMB2A -14	1.81	0.055	61942.46	113493.35	61942.46	0.575	2.2	18.066	3.9	0.228	3.3	0.8	1322	39	2993	39	4447	32
SMB2A -15	1.43	0.074	59218.65	101957.12	59218.65	0.613	3.0	21.842	6.9	0.259	6.2	0.9	1483	83	3177	69	4538	43
SMB2A -16	2.18	0.192	110934.43	180665.93	110934.43	0.647	5.7	26.829	11.5	0.301	10.0	0.9	1695	151	3377	120	4617	83
SMB2A -17	2.50	0.006	56256.23	124936.80	56256.23	0.475	1.1	11.869	2.5	0.181	2.2	0.9	1074	22	2594	24	4165	16
SMB2A -18	2.39	0.134	72997.56	141942.24	72997.56	0.542	1.2	16.111	4.0	0.216	3.8	1.0	1258	44	2883	39	4360	18
SMB2A -19	1.59	0.018	46221.24	92666.60	46221.24	0.526	1.4	15.338	3.0	0.211	2.6	0.9	1236	29	2836	29	4317	21
SMB2A -20	1.33	0.083	84651.05	127390.57	84651.05	0.700	4.4	33.707	17.0	0.349	16.4	1.0	1930	280	3602	183	4731	63
SMB2A -21	1.42	-0.004	47627.62	89033.78	47627.62	0.564	1.2	17.697	2.6	0.227	2.3	0.9	1321	27	2973	25	4419	18
SMB2A -22	1.36	-0.011	39753.05	79634.30	39753.05	0.527	1.1	15.449	2.3	0.213	2.0	0.9	1243	23	2843	23	4318	17
SMB2A -23	1.34	0.006	40150.49	79169.99	40150.49	0.535	0.8	15.938	4.6	0.216	4.5	1.0	1260	52	2873	45	4342	12
SMB2A -24	0.46	0.107	82663.09	104361.29	82663.09	0.835	1.0	94.824	5.6	0.824	5.5	1.0	3874	161	4633	57	4982	14
SMB2A -25	1.31	0.052	88343.82	133023.75	88343.82	0.700	2.0	35.925	5.6	0.372	5.3	0.9	2040	93	3664	57	4730	29
SMB2A -26	2.58	0.044	74340.42	148665.39	74340.42	0.527	1.6	15.317	3.7	0.211	3.4	0.9	1233	38	2835	36	4319	24
SMB2A -27	1.63	0.068	69883.68	119590.42	69883.68	0.616	2.1	22.736	6.3	0.268	6.0	0.9	1529	82	3216	64	4546	30
SMB2A -28	1.51	0.020	63328.31	108045.84	63328.31	0.618	0.7	22.311	1.9	0.262	1.8	0.9	1499	24	3197	19	4551	9
SMB2A -29	1.53	0.014	51981.43	96586.96	51981.43	0.568	0.8	18.027	2.4	0.230	2.3	0.9	1336	27	2991	23	4427	12
SMB2A -30	0.65	0.214	102261.91	129803.25	102261.91	0.830	0.7	83.605	3.6	0.731	3.5	1.0	3535	97	4506	37	4974	9
SMB2A -31	1.84	-0.001	38397.37	90433.23	38397.37	0.449	2.2	11.136	3.7	0.180	3.0	0.8	1067	29	2535	35	4080	33
SMB2A -32	0.44	0.703	67321.37	85654.56	67321.37	0.828	0.6	81.919	2.8	0.717	2.7	1.0	3486	74	4486	29	4971	8
SMB2A -33	0.49	0.611	69109.19	88440.80	69109.19	0.825	0.9	75.929	4.3	0.668	4.2	1.0	3297	110	4410	44	4965	13
SMB2A -34	0.53	0.590	75829.14	96976.26	75829.14	0.824	1.0	76.974	11.9	0.678	11.8	1.0	3335	315	4423	126	4964	14
SMB2A -35	0.44	1.164	90148.21	112244.41	90148.21	0.846	0.9	109.136	2.8	0.936	2.7	1.0	4258	85	4774	29	5001	12
SMB2A -36	0.39	0.812	76304.69	93551.18	76304.69	0.859	1.1	105.674	5.4	0.892	5.3	1.0	4110	163	4742	56	5023	16
SMB2A -37	0.38	0.737	78656.33	95601.12	78656.33	0.867	0.8	109.642	8.2	0.918	8.1	1.0	4197	256	4779	86	5035	12
SMB2A -38	0.63	0.772	69235.45	92093.66	69235.45	0.794	0.6	58.816	3.5	0.537	3.5	1.0	2771	78	4154	36	4912	9
SMB2A -39	0.52	0.952	81269.41	103976.47	81269.41	0.824	1.3	84.225	4.4	0.742	4.2	1.0	3577	117	4514	45	4963	19

SAMBRA QUARRY - SMB2B (micrite matrix) - Pedro Leopoldo cap carbonate member

SPOT	U(ppm)	Th/U	207Pb(cps)	206Pb(cps)	208Pb(cps)	207Pb/206Pb	2sigma(%)	207Pb/235U	2sigma(%)	206Pb/238U	2sigma(%)	Rho	206Pb/238U	2sigma(s)	207Pb/235U	2sigma(s)	207Pb/206Pb	2sigma(s)

SMB2B - 1	1.49	0.062	102423.79	149166.81	212682.09	0.740	1.5	47.375	8.8	0.464	8.7	1.0	2459	180	3939	91	4810	22
SMB2B - 2	1.03	0.014	53112.90	84561.28	112526.94	0.716	0.8	37.703	3.0	0.382	2.9	1.0	2086	51	3712	30	4763	11
SMB2B - 3	0.72	0.006	64764.56	85586.50	139359.77	0.793	0.7	60.178	2.6	0.550	2.5	1.0	2827	58	4177	26	4909	10
SMB2B - 4	2.20	-0.013	38098.08	87234.70	76517.78	0.463	1.8	11.788	2.9	0.185	2.3	0.8	1092	23	2588	28	4128	27
SMB2B - 5	2.12	0.012	78227.79	127325.32	162764.81	0.640	2.0	24.700	4.1	0.280	3.6	0.9	1590	52	3296	41	4602	28
SMB2B - 6	2.08	-0.010	31107.04	75796.03	61830.46	0.439	1.1	10.269	2.2	0.170	1.9	0.9	1011	18	2459	21	4047	17
SMB2B - 7	7.15	-0.002	38098.56	185377.67	61857.07	0.220	1.4	3.669	2.3	0.121	1.9	0.8	736	13	1565	19	2982	22
SMB2B - 8	2.60	-0.011	39552.16	95027.52	78672.36	0.436	1.8	10.264	3.3	0.171	2.8	0.8	1017	26	2459	31	4037	27
SMB2B - 9	3.58	-0.006	31201.44	105611.53	57392.78	0.310	2.2	5.880	3.1	0.138	2.1	0.7	832	17	1958	27	3519	35
SMB2B - 10	2.19	-0.005	26919.96	72701.81	52536.20	0.387	0.8	8.273	2.4	0.155	2.2	0.9	929	19	2261	22	3860	13
SMB2B - 11	2.86	-0.010	42125.41	101968.58	83407.58	0.431	1.7	9.899	3.0	0.167	2.5	0.8	994	23	2425	28	4020	26
SMB2B - 12	2.61	-0.003	43061.49	100083.39	85678.53	0.448	2.8	11.100	3.8	0.180	2.6	0.7	1065	26	2532	36	4080	41
SMB2B - 13	2.10	-0.009	23550.05	67505.90	44784.05	0.367	1.8	7.604	2.8	0.150	2.2	0.8	903	19	2185	26	3779	27

TATIANA QUARRY - TATIA (crystal-fans) - Lagoa Santa Member, base

SPOT	U(ppm)	Th/U	207Pb(cps)	206Pb(cps)	208Pb(cps)	207Pb/206Pb	2sigma(%)	207Pb/235U	2sigma(%)	206Pb/238U	2sigma(%)	Rho	206Pb/238U	2sigma(s)	207Pb/235U	2sigma(s)	207Pb/206Pb	2sigma(s)
TATIA - 1	2.29	-0.007	32822.29	83087.19	32822.29	0.448	5.4	10.584	8.7	0.171	6.8	0.8	1020	64	2487	84	4078	81
TATIA - 2	1.24	-0.009	29147.17	59829.16	29147.17	0.552	2.8	17.358	5.5	0.228	4.8	0.9	1324	57	2955	54	4387	41
TATIA - 3	1.64	-0.007	23273.20	59890.61	23273.20	0.441	1.6	10.495	2.3	0.173	1.6	0.7	1027	16	2479	22	4054	24
TATIA - 4	1.32	-0.004	28488.79	60738.57	28488.79	0.530	0.6	15.831	1.3	0.216	1.2	0.9	1263	14	2867	13	4328	9
TATIA - 5	1.61	-0.002	31570.64	67995.95	31570.64	0.519	0.6	14.415	1.3	0.201	1.2	0.9	1182	13	2777	13	4297	9
TATIA - 6	1.55	-0.010	25622.91	60937.99	25622.91	0.477	1.3	12.148	2.3	0.185	2.0	0.8	1093	20	2616	22	4170	19
TATIA - 7	1.58	-0.013	23662.61	58464.95	23662.61	0.459	1.5	11.027	2.0	0.174	1.3	0.7	1035	13	2525	19	4115	22
TATIA - 8	1.92	-0.009	28033.28	71346.82	28033.28	0.445	3.2	10.767	5.3	0.175	4.1	0.8	1042	40	2503	50	4069	48
TATIA - 9	1.73	-0.004	34699.42	75757.02	34699.42	0.518	3.2	14.714	8.3	0.206	7.6	0.9	1208	84	2797	82	4293	47
TATIA - 10	1.49	0.020	78095.91	125318.83	78095.91	0.705	2.8	38.406	13.6	0.395	13.3	1.0	2146	248	3730	145	4741	41
TATIA - 11	1.33	0.035	45537.15	82504.59	45537.15	0.625	1.5	25.083	3.8	0.291	3.5	0.9	1647	51	3311	38	4567	22
TATIA - 12	1.28	-0.011	34180.59	66555.61	34180.59	0.581	1.2	19.705	3.2	0.246	2.9	0.9	1417	38	3077	31	4462	18
TATIA - 13	1.36	-0.004	23309.43	61198.25	23309.43	0.583	1.8	19.763	3.0	0.246	2.4	0.8	1417	31	3080	29	4467	26
TATIA - 14	1.93	0.004	33214.45	75771.41	33214.45	0.496	1.0	12.666	1.6	0.185	1.3	0.8	1095	13	2655	16	4230	15
TATIA - 15	1.30	-0.005	37643.37	70391.04	37643.37	0.605	1.0	21.146	2.7	0.253	2.5	0.9	1456	32	3145	26	4520	15
TATIA - 16	2.18	-0.008	29310.12	76533.97	29310.12	0.433	1.1	9.827	1.7	0.165	1.3	0.8	982	12	2419	16	4028	17
TATIA - 17	3.61	-0.005	53319.10	134039.45	53319.10	0.450	0.8	10.824	1.8	0.174	1.6	0.9	1036	16	2508	17	4085	12
TATIA - 18	3.99	0.000	42411.47	127119.60	42411.47	0.376	2.8	7.785	3.8	0.150	2.5	0.7	902	21	2207	35	3816	43
TATIA - 19	2.64	-0.011	23821.91	77743.44	23821.91	0.346	1.6	6.610	2.1	0.138	1.4	0.7	835	11	2061	19	3692	24
TATIA - 20	3.92	-0.003	29851.66	110185.09	29851.66	0.307	1.5	5.581	2.5	0.132	2.0	0.8	799	15	1913	22	3505	23
TATIA - 21	2.34	-0.011	21336.20	70679.74	21336.20	0.342	1.4	6.686	1.8	0.142	1.2	0.6	855	9	2071	16	3671	22

TATIANA QUARRY - TATIB (micrite matrix) - Lagoa Santa Member, base

SPOT	U(ppm)	Th/U	207Pb(cps)	206Pb(cps)	208Pb(cps)	207Pb/206Pb	2sigma(%)	207Pb/235U	2sigma(%)	206Pb/238U	2sigma(%)	Rho	206Pb/238U	2sigma(s)	207Pb/235U	2sigma(s)	207Pb/206Pb	2sigma(s)
TATIB - 1	0.62	1.513	104109.24	123554.48	222537.20	0.873	0.6	141.057	8.8	1.172	8.7	1.0	5000	311	5032	92	5046	9
TATIB - 2	0.41	1.932	73601.91	89074.60	158993.01	0.856	0.7	149.488	3.7	1.266	3.6	1.0	5274	133	5091	38	5018	10
TATIB - 3	0.59	2.319	68634.10	84986.11	149874.87	0.837	0.7	98.226	4.9	0.851	4.9	1.0	3970	146	4668	51	4986	10
TATIB - 4	0.51	1.553	88865.82	104274.75	190534.77	0.883	0.5	145.098	7.7	1.192	7.7	1.0	5058	274	5061	81	5062	8
TATIB - 5	0.58	2.072	67887.85	83435.88	147769.62	0.843	0.7	97.125	5.2	0.836	5.1	1.0	3916	152	4657	53	4996	9
TATIB - 6	0.34	1.050	82360.13	97370.06	175952.91	0.876	0.6	206.504	4.0	1.709	4.0	1.0	6424	163	5417	41	5051	8
TATIB - 7	0.39	2.155	75786.46	89784.29	163335.22	0.875	0.6	160.850	4.0	1.334	4.0	1.0	5463	148	5165	41	5048	8
TATIB - 8	0.54	4.277	61482.61	75946.00	138567.75	0.839	0.6	96.181	3.4	0.831	3.4	1.0	3900	99	4647	35	4990	8
TATIB - 9	0.47	2.184	79230.07	94610.97	171064.47	0.868	0.6	141.535	6.4	1.183	6.4	1.0	5032	228	5036	67	5037	9
TATIB - 10	0.44	0.961	56966.47	69426.14	122094.32	0.851	0.9	109.131	6.0	0.930	5.9	1.0	4238	187	4774	62	5010	12
TATIB - 11	0.39	2.277	107455.29	124999.11	229126.22	0.890	0.5	231.410	4.3	1.885	4.3	1.0	6830	182	5532	44	5074	7
TATIB - 12	0.41	1.447	92428.13	108445.05	197298.87	0.883	0.5	188.982	2.8	1.552	2.8	1.0	6041	110	5328	29	5062	7
TATIB - 13	0.50	2.049	81442.77	96875.86	175456.27	0.871	0.6	137.416	7.5	1.144	7.4	1.0	4917	261	5006	78	5043	9
TATIB - 14	0.47	1.862	93200.66	109788.60	199643.82	0.879	0.6	167.445	4.9	1.381	4.9	1.0	5593	186	5205	51	5056	8
TATIB - 15	2.51	-0.011	11039.32	49045.20	18209.98	0.235	2.6	3.710	3.2	0.115	1.9	0.6	700	13	1574	26	3085	41
TATIB - 16	0.72	-0.034	16883.91	29552.46	34468.95	0.594	1.7	19.688	3.2	0.240	2.7	0.8	1388	33	3076	31	4494	24
TATIB - 17	2.15	-0.014	19318.12	53560.02	36148.64	0.375	3.0	7.544	3.6	0.146	1.9	0.5	878	16	2178	33	3812	46
TATIB - 18	0.72	-0.020	15008.22	27163.06	30497.64	0.575	1.4	17.549	2.7	0.221	2.3	0.9	1289	27	2965	27	4446	20
TATIB - 19	0.48	-0.015	16591.33	25636.27	34408.41	0.673	1.8	28.971	4.6	0.312	4.2	0.9	1752	65	3453	46	4674	25
TATIB - 20	2.35	-0.010	21466.00	58546.94	40591.91	0.381	2.4	7.667	3.4	0.146	2.5	0.7	879	20	2193	31	3835	36

ROAD POLICE STATION - RP 1 (dark stromatolites) - Lagoa Santa Member, top

SPOT	U(ppm)	Th/U	207Pb(cps)	206Pb(cps)	208Pb(cps)	207Pb/206Pb	2sigma(%)	207Pb/235U	2sigma(%)	206Pb/238U	2sigma(%)	Rho	206Pb/238U	2sigma(s)	207Pb/235U	2sigma(s)	207Pb/206Pb	2sigma(s)
RP1 - 3	0.68	-0.037	6268.44	26271.96	6268.44	0.726	2.4	42.930	8.8	0.429	8.5	1.0	2301	167	3841	92	4782	35
RP1 - 4	1.40	0.020	22197.50	43098.27	22197.50	0.638	1.4	24.926	3.4	0.283	3.1	0.9	1608	44	3305	34	4598	21
RP1 - 5	2.38	-0.009	17906.31	45827.29	17906.31	0.486	0.5	11.911	3.0	0.178	2.9	1.0	1054	28	2597	28	4200	7
RP1 - 6	1.77	-0.003	17508.53	40523.62	17508.53	0.534	1.7	15.573	3.8	0.211	3.3	0.9	1236	38	2851	37	4339	26
RP1 - 7	2.22	-0.010	13319.59	39697.74	13319.59	0.429	3.2	9.754	5.0	0.165	3.8	0.8	984	35	2412	47	4013	48

RP1 - 8	1.61	-0.020	27746.48	53016.20	27746.48	0.654	3.0	27.523	5.7	0.305	4.9	0.9	1717	74	3402	58	4633	44
RP1 - 9	1.53	0.091	30392.75	54978.22	30392.75	0.680	1.1	31.232	3.3	0.333	3.1	0.9	1854	51	3526	33	4688	16
RP1 - 10	2.22	0.006	19295.28	45665.47	19295.28	0.524	0.7	13.770	3.0	0.191	3.0	1.0	1126	31	2734	29	4309	10
RP1 - 11	2.63	-0.002	21059.99	52048.14	21059.99	0.496	1.0	12.526	3.1	0.183	3.0	0.9	1085	30	2645	30	4228	15
RP1 - 12	1.02	0.021	26860.13	45419.68	26860.13	0.723	0.5	41.137	3.1	0.412	3.0	1.0	2226	57	3798	31	4778	8
RP1 - 13	2.15	-0.008	24140.91	52290.96	24140.91	0.570	1.0	17.776	3.2	0.226	3.1	1.0	1314	36	2978	31	4433	14
RP1 - 14	6.74	-0.004	21557.25	92599.82	21557.25	0.289	4.2	5.091	6.1	0.128	4.3	0.7	774	32	1835	53	3414	66
RP1 - 15	2.55	-0.004	36523.43	73983.06	36523.43	0.606	3.6	22.535	8.8	0.270	8.1	0.9	1539	111	3207	90	4523	52
RP1 - 16	5.77	-0.005	5234.89	49475.80	5234.89	0.330	8.9	6.201	11.6	0.136	7.4	0.6	824	58	2005	107	3617	136
RP1 - 17	2.75	-0.005	24948.23	58055.21	24948.23	0.523	0.8	14.195	3.1	0.197	3.0	1.0	1158	32	2763	30	4308	12
RP1 - 18	2.67	-0.006	23241.13	55629.59	23241.13	0.506	0.5	13.558	2.9	0.194	2.9	1.0	1146	30	2719	28	4258	7
RP1 - 19	3.45	-0.008	26814.10	68221.78	26814.10	0.480	2.1	12.235	4.3	0.185	3.8	0.9	1093	38	2623	41	4182	31
RP1 - 20	4.17	-0.004	26602.77	73456.94	26602.77	0.441	3.4	10.006	5.5	0.165	4.3	0.8	983	39	2435	52	4054	50
RP1 - 21	2.31	-0.010	22851.53	52098.74	22851.53	0.532	1.3	15.512	3.8	0.211	3.6	0.9	1236	41	2847	37	4334	20
RP1 - 22	2.39	-0.008	24533.17	54974.21	24533.17	0.543	1.5	16.142	4.5	0.216	4.3	0.9	1260	49	2885	44	4361	22
RP1 - 23	2.96	-0.005	24944.33	58584.40	24944.33	0.514	1.0	13.196	3.1	0.186	2.9	0.9	1100	30	2694	30	4283	14
RP1 - 24	2.56	-0.020	29913.86	61175.46	29913.85	0.590	0.5	18.292	3.0	0.225	2.9	1.0	1307	35	3005	29	4484	7
RP1 - 25	2.65	-0.009	53875.33	96560.86	53875.33	0.673	1.0	31.892	3.2	0.344	3.0	1.0	1904	50	3547	32	4675	14

Reference materials

SPOT	U(ppm)	Th/U	207Pb(cps)	206Pb(cps)	208Pb(cps)	207Pb/206Pb	2sigma(%)	207Pb/235U	2sigma(%)	206Pb/238U	2sigma(%)	Rho	206Pb/238U	2sigma(s)	207Pb/235U	2sigma(s)	207Pb/206Pb	2sigma(s)
**NAMIBIA**																		
NAM-1	1.53	0.027	20827.06	55421.88	20827.06	0.430	1.2	8.949	1.9	0.151	1.4	0.8	906	12	2333	17	4019	18
NAM-2	1.29	0.018	19556.01	49492.38	19556.01	0.453	1.1	9.953	1.7	0.160	1.3	0.8	954	12	2430	16	4094	17
NAM-3	1.30	0.011	21983.01	52159.46	21983.01	0.482	0.9	11.101	1.5	0.167	1.2	0.8	995	11	2532	14	4188	13
NAM-4	1.11	-0.010	27352.67	55819.96	27352.67	0.561	1.7	16.238	2.7	0.210	2.0	0.8	1229	23	2891	26	4410	25
NAM-5	1.48	-0.004	16429.26	49476.33	16429.26	0.380	0.9	7.335	1.5	0.140	1.2	0.8	844	9	2153	14	3833	14
NAM-6	1.42	0.050	40504.55	77126.40	40504.55	0.601	0.8	18.735	1.7	0.226	1.5	0.9	1314	18	3028	17	4510	11
NAM-7	0.85	-0.001	14615.18	34790.03	14615.18	0.481	1.2	11.384	1.8	0.172	1.3	0.7	1021	12	2555	17	4185	18
NAM-8	1.80	0.011	38402.75	82242.41	38402.75	0.534	1.0	14.071	1.8	0.191	1.5	0.8	1127	15	2755	17	4339	14
**NAMIBIA**																		
NAM-1	1.39	0.030	20777.29	56189.58	20777.29	0.402	2.7	8.079	3.1	0.146	1.6	0.5	878	13	2240	29	3915	41
NAM-2	1.19	0.017	19991.59	50647.24	19991.59	0.429	1.1	9.086	1.7	0.154	1.3	0.7	922	11	2347	16	4013	17
NAM-3	1.23	0.011	23122.75	55630.00	23122.75	0.451	1.7	10.211	2.3	0.164	1.5	0.7	980	14	2454	21	4089	25
NAM-4	1.01	-0.010	27352.67	55819.96	27352.67	0.532	1.8	14.708	2.8	0.201	2.1	0.8	1178	23	2797	27	4332	27
NAM-5	1.32	-0.009	16254.36	49484.13	16254.36	0.357	1.8	6.690	2.2	0.136	1.4	0.6	822	11	2071	20	3736	27
NAM-6	1.32	0.044	44424.41	82436.56	44424.41	0.585	0.9	18.304	1.8	0.227	1.5	0.9	1319	18	3006	17	4470	13
NAM-7	0.77	-0.004	14697.81	34907.55	14697.81	0.458	1.2	10.348	1.8	0.164	1.3	0.7	979	12	2466	17	4110	18

SPOT	U(ppm)	Th/U	207Pb(cps)	206Pb(cps)	208Pb(cps)	207Pb/206Pb	2sigma(%)	207Pb/235U	2sigma(%)	206Pb/238U	2sigma(%)	Rho	206Pb/238U	2sigma(s)	207Pb/235U	2sigma(s)	207Pb/206Pb	2sigma(s)
**WC**																		
WC - 1	4.27	-0.004	6994.20	55530.63	6994.20	0.132	1.6	0.809	2.0	0.045	1.2	0.6	281	3	602	9	2121	28
WC - 2	4.07	-0.004	7698.69	53571.93	7698.69	0.141	8.3	0.877	8.4	0.045	1.2	0.1	284	3	639	40	2240	143
WC - 3	3.83	-0.006	5381.63	48335.80	5381.63	0.110	2.0	0.654	2.3	0.043	1.2	0.5	273	3	511	9	1794	37
WC - 4	4.13	-0.004	6124.80	51319.46	6124.80	0.118	2.4	0.716	2.7	0.044	1.3	0.5	277	3	548	11	1927	42
WC - 5	4.72	-0.004	11325.10	64447.66	11325.10	0.172	6.4	1.111	6.6	0.047	1.7	0.3	295	5	759	36	2578	107
WC - 6	5.71	-0.002	9037.26	72949.61	9037.26	0.121	5.6	0.734	5.7	0.044	1.3	0.2	276	4	559	25	1978	99
WC - 7	4.97	-0.004	7822.52	63171.86	7822.52	0.122	7.1	0.732	7.3	0.044	1.5	0.2	275	4	557	32	1980	127
WC - 8	4.69	-0.005	6994.20	55530.63	6994.20	0.160	1.3	1.019	1.8	0.046	1.2	0.7	292	3	713	9	2452	23
WC - 9	4.47	-0.004	7698.69	53571.93	7698.69	0.171	6.8	1.104	6.9	0.047	1.2	0.2	295	4	755	38	2566	114
WC - 10	4.06	-0.003	12889.49	57277.02	12889.49	0.267	8.9	2.032	10.3	0.055	5.1	0.5	346	17	1126	73	3289	140
WC - 11	4.21	-0.006	5381.63	48335.80	5381.63	0.133	1.7	0.824	2.1	0.045	1.2	0.6	284	3	610	10	2137	29
WC - 12	4.51	-0.005	6112.75	51244.63	6112.75	0.143	1.5	0.906	1.9	0.046	1.2	0.6	289	3	655	9	2264	25
WC - 13	5.53	-0.005	9073.82	64982.80	9073.82	0.166	2.1	1.054	2.4	0.046	1.2	0.5	290	3	731	13	2517	34
WC - 14	6.22	-0.002	8425.74	71028.30	8425.74	0.141	2.6	0.869	2.8	0.045	1.2	0.4	282	3	635	14	2239	44
WC - 15	5.46	-0.004	7822.52	63171.86	7822.52	0.147	5.9	0.922	6.1	0.045	1.5	0.2	286	4	663	30	2316	101
WC - 16	4.30	0.001	16172.54	62518.51	16172.54	0.307	6.3	2.516	6.8	0.059	2.4	0.3	372	9	1277	50	3507	98
WC - 17	3.78	-0.007	9922.88	48987.86	9922.88	0.242	4.0	1.764	4.2	0.053	1.5	0.4	333	5	1032	28	3130	63
WC - 18	5.58	-0.002	7505.16	62774.52	7505.16	0.143	3.0	0.905	3.2	0.046	1.3	0.4	290	4	654	16	2258	51
WC - 19	5.01	0.000	16038.91	68858.63	16038.91	0.276	8.8	2.145	9.3	0.056	3.0	0.3	353	10	1163	66	3342	138
WC - 20	3.65	-0.004	9024.80	46344.97	9024.80	0.232	4.9	1.666	5.1	0.052	1.6	0.3	327	5	996	33	3068	78
WC - 21	5.51	-0.004	10987.00	65896.77	10987.00	0.199	1.0	1.342	1.6	0.049	1.2	0.8	309	4	864	9	2814	16
WC - 22	6.60	-0.001	8928.25	73701.44	8928.25	0.144	2.4	0.912	2.7	0.046	1.2	0.5	289	3	658	13	2281	41
WC - 23	5.56	-0.004	5084.85	58697.88	5084.85	0.105	1.6	0.627	2.0	0.043	1.2	0.6	274	3	494	8	1712	30
WC - 24	6.01	-0.003	6776.61	53469.06	9186.23	0.165	5.7	1.066	6.4	0.047	2.9	0.5	295	8	737	34	2507	95
WC - 25	4.60	-0.005	12464.17	51162.77	22747.51	0.309	12.2	2.496	13.2	0.059	5.0	0.4	367	18	1271	100	3516	188

WC - 26	3.58	-0.009	4875.11	33162.10	6807.59	0.190	6.1	1.279	6.7	0.049	2.8	0.4	307	9	836	39	2744	100
WC - 27	5.40	-0.005	4440.11	45210.56	4440.37	0.128	5.3	0.780	6.0	0.044	2.9	0.5	279	8	586	27	2072	93
WC - 28	4.55	-0.006	4004.30	38635.94	4724.92	0.148	8.6	0.912	9.1	0.045	2.9	0.3	283	8	658	45	2318	147
WC - 29	4.90	-0.004	5970.36	43241.14	7916.89	0.178	4.5	1.143	5.4	0.047	2.9	0.5	294	8	774	29	2632	75
WC - 30	8.14	-0.001	23542.88	89422.36	43955.15	0.312	2.6	2.500	4.0	0.058	3.0	0.8	364	11	1272	29	3533	40
WC - 31	5.66	-0.003	5017.99	47806.06	5372.26	0.131	7.4	0.807	7.9	0.045	2.8	0.4	281	8	601	36	2115	129
**WC**																		
WC - 1	5.61	-0.005	4983.16	41374.36	50669.86	0.117	3.0	0.706	4.1	0.044	2.8	0.7	276	8	542	17	1908	54
WC - 2	5.37	-0.006	7435.83	52940.82	51688.49	0.159	12.4	1.028	12.9	0.047	3.5	0.3	296	10	718	69	2441	210
WC - 3	3.31	-0.008	4733.19	32600.34	31966.64	0.159	6.5	1.031	7.1	0.047	2.8	0.4	296	8	720	37	2450	110
WC - 4	5.05	-0.008	4794.36	46273.46	45760.75	0.112	3.3	0.674	4.4	0.044	2.9	0.7	276	8	523	18	1829	60
WC - 5	4.16	-0.008	4123.30	38607.97	38083.08	0.126	4.4	0.773	5.3	0.045	2.9	0.5	281	8	582	24	2038	78
WC - 6	4.76	-0.006	6145.34	45284.71	44707.73	0.145	1.5	0.905	3.2	0.045	2.8	0.9	286	8	654	16	2282	27
WC - 7	8.22	-0.001	22389.78	91083.40	91714.29	0.244	3.8	1.799	4.9	0.053	3.1	0.6	336	10	1045	32	3146	60
WC - 8	5.22	-0.003	6066.33	48486.59	48115.62	0.130	7.5	0.792	8.1	0.044	3.0	0.4	279	8	592	37	2094	133
WC - 9	4.46	-0.015	1287.08	14792.36	1287.08	0.101	2.4	0.606	4.5	0.044	3.8	0.8	275	10	481	17	1637	45
WC - 10	4.91	-0.011	5686.39	21210.94	5686.39	0.272	15.6	2.136	16.9	0.057	6.4	0.4	357	22	1160	124	3318	244
WC - 11	5.79	-0.011	8460.74	26912.09	8460.74	0.317	10.8	2.682	12.9	0.061	7.0	0.5	383	26	1323	100	3557	167
WC - 12	6.42	-0.002	2710.48	22125.19	2710.48	0.131	3.7	0.819	5.3	0.045	3.8	0.7	287	11	607	25	2105	64
WC - 13	4.77	-0.013	2546.84	17326.07	2546.84	0.174	1.9	1.155	4.2	0.048	3.8	0.9	304	11	780	23	2592	32
WC - 14	6.12	-0.009	2866.73	21496.55	2866.73	0.149	3.4	0.953	5.1	0.046	3.8	0.7	293	11	680	25	2331	58
WC - 15	3.07	-0.024	6611.38	16989.62	6611.38	0.406	10.6	4.099	14.9	0.073	10.5	0.7	456	46	1654	130	3930	160
WC - 16	5.76	-0.011	2455.05	19779.70	2455.05	0.136	1.8	0.850	4.2	0.045	3.8	0.9	287	11	625	20	2173	31
WC - 17	8.20	-0.007	6519.60	31858.23	6519.60	0.214	1.6	1.521	4.1	0.052	3.8	0.9	324	12	939	26	2936	26
WC - 18	8.84	-0.007	4778.71	30637.50	4778.71	0.165	2.4	1.078	4.5	0.047	3.8	0.8	298	11	742	24	2509	41
WC - 19	9.19	-0.009	4501.59	31203.88	4501.59	0.153	2.6	0.979	4.6	0.046	3.8	0.8	292	11	693	23	2382	44
WC - 20	10.58	-0.005	3218.20	33700.92	3218.20	0.111	2.2	0.669	4.3	0.044	3.7	0.9	275	10	520	18	1821	40
WC - 21	10.50	-0.006	5092.76	35513.40	5092.76	0.158	2.1	1.008	4.3	0.046	3.7	0.9	292	11	708	22	2433	36
WC - 22	7.71	-0.009	4187.57	26421.69	4187.57	0.164	1.8	1.063	4.1	0.047	3.7	0.9	296	11	735	22	2497	30
**SPOT**	**U(ppm)**	**Th/U**	**207Pb(cps)**	**206Pb(cps)**	**208Pb(cps)**	**207Pb/206Pb**	**2sigma(%)**	**207Pb/235U**	**2sigma(%)**	**206Pb/238U**	**2sigma(%)**	**Rho**	**206Pb/238U**	**2sigma(s)**	**207Pb/235U**	**2sigma(s)**	**207Pb/206Pb**	**2sigma(s)**
**DB**																		
DB - 1	2.23	-0.013	47.41	5294.19	-363.56	0.172	4.2	0.276	4.6	0.012	1.9	0.4	75	1	248	10	2579	71
DB - 2	2.59	-0.010	2073.75	9890.56	3937.82	0.440	3.8	1.139	5.0	0.019	3.2	0.6	120	4	772	27	4051	57
DB - 3	1.75	-0.013	-1442.23	4600.07	-3584.84	0.279	4.4	0.498	5.0	0.013	2.3	0.5	83	2	410	17	3359	69
DB - 4	2.68	-0.012	-1068.40	7927.95	-2766.88	0.313	4.2	0.629	4.7	0.015	2.2	0.5	93	2	496	19	3536	65
DB - 5	1.32	-0.022	641.19	5323.56	1082.03	0.461	2.6	1.260	3.6	0.020	2.4	0.7	127	3	828	20	4121	39
DB - 6	1.77	-0.017	108.30	5412.49	-156.65	0.333	4.4	0.693	5.1	0.015	2.4	0.5	97	2	535	21	3630	68
DB - 7	1.46	-0.021	-1015.28	4447.82	-2497.30	0.326	5.4	0.676	6.0	0.015	2.7	0.5	96	3	524	25	3600	82
DB - 8	1.89	-0.019	570.44	6098.30	797.63	0.354	3.7	0.777	4.4	0.016	2.4	0.5	102	2	584	20	3724	56
DB - 9	3.56	-0.009	1273.03	10094.43	1963.00	0.284	3.9	0.547	4.5	0.014	2.2	0.5	89	2	443	16	3386	61
DB - 10	2.70	-0.013	850.14	7562.00	1410.99	0.274	4.3	0.521	4.8	0.014	2.1	0.4	88	2	426	17	3329	68
DB - 11	2.05	-0.018	743.77	6013.59	1125.36	0.304	4.3	0.606	5.1	0.014	2.8	0.6	93	3	481	20	3489	66
**DB**																		
DB - 1	2.57	-0.009	450.25	5681.89	415.03	0.122	4.0	0.186	4.4	0.011	1.8	0.4	71	1	173	7	1991	71
DB - 2	2.18	-0.015	507.95	4936.80	626.34	0.137	3.7	0.214	4.1	0.011	1.9	0.5	72	1	197	7	2194	64
DB - 3	2.68	-0.011	913.05	6382.69	1445.83	0.186	11.8	0.306	12.4	0.012	3.9	0.3	76	3	271	30	2710	195
DB - 4	1.02	-0.026	539.12	2725.67	998.80	0.258	6.5	0.477	7.0	0.013	2.7	0.4	86	2	396	23	3237	102
DB - 5	2.39	-0.013	703.39	5412.27	1004.16	0.156	5.2	0.243	5.5	0.011	1.9	0.4	72	1	221	11	2413	88
DB - 6	1.29	-0.023	3748.00	6475.84	7927.70	0.576	9.6	2.001	17.9	0.025	15.1	0.8	160	24	1116	129	4449	140
**SPOT**	**U(ppm)**	**Th/U**	**207Pb(cps)**	**206Pb(cps)**	**208Pb(cps)**	**207Pb/206Pb**	**2sigma(%)**	**207Pb/235U**	**2sigma(%)**	**206Pb/238U**	**2sigma(%)**	**Rho**	**206Pb/238U**	**2sigma(s)**	**207Pb/235U**	**2sigma(s)**	**207Pb/206Pb**	**2sigma(s)**
**RM**																		
RM - 1	2.91	-0.009	525.71	6433.51	525.71	0.112	3.1	0.164	3.3	0.011	1.1	0.3	68	1	154	5	1826	56
RM - 2	1.69	-0.015	378.83	3690.07	378.83	0.134	4.0	0.201	4.2	0.011	1.2	0.3	70	1	186	7	2145	70
RM - 3	2.45	-0.010	411.87	5317.23	411.87	0.108	3.8	0.155	3.9	0.010	1.2	0.3	67	1	146	5	1760	69
RM - 4	1.89	-0.015	333.96	4213.06	333.96	0.123	3.8	0.182	4.0	0.011	1.2	0.3	69	1	170	6	2006	68
RM - 5	1.89	-0.016	365.30	4209.77	365.30	0.123	3.4	0.181	3.6	0.011	1.2	0.3	69	1	169	6	1996	60
RM - 6	2.55	-0.011	405.18	5475.54	405.18	0.096	3.4	0.136	3.6	0.010	1.2	0.3	66	1	130	4	1542	64
RM - 7	2.02	-0.015	355.78	4378.69	355.78	0.106	4.1	0.153	4.2	0.010	1.2	0.3	67	1	144	6	1736	75
RM - 8	2.74	-0.011	438.43	5810.41	438.43	0.097	3.5	0.136	3.7	0.010	1.2	0.3	66	1	130	5	1564	66
**RM**																		
**SPOT**	**U(ppm)**	**Th/U**	**207Pb(cps)**	**206Pb(cps)**	**208Pb(cps)**	**207Pb/206Pb**	**2sigma(%)**	**207Pb/235U**	**2sigma(%)**	**206Pb/238U**	**2sigma(%)**	**Rho**	**206Pb/238U**	**2sigma(s)**	**207Pb/235U**	**2sigma(s)**	**207Pb/206Pb**	**2sigma(s)**
RM - 1	0.51	-0.117	200.07	1174.90	200.07	0.634	3.9	2.699	6.8	0.031	5.6	0.8	196	11	1328	52	4589	56
RM - 2	0.27	-0.227	66.48	810.30	66.48	0.683	3.6	3.707	6.4	0.039	5.2	0.8	249	13	1573	52	4695	52

RM - 3	0.44	-0.141	113.47	1271.57	113.47	0.674	3.2	3.556	6.0	0.038	5.1	0.8	242	12	1540	49	4677	46
RM - 4	0.32	-0.209	247.54	1059.80	247.54	0.687	3.2	4.062	6.0	0.043	5.1	0.9	271	14	1647	51	4704	46
RM - 5	0.32	-0.245	331.14	1008.92	331.14	0.696	3.0	4.000	5.9	0.042	5.1	0.9	263	13	1634	49	4723	43
RM - 6	0.42	-0.161	81.98	714.10	81.98	0.568	3.8	2.032	6.1	0.026	4.7	0.8	165	8	1126	42	4428	56
RM - 7	0.29	-0.216	93.67	858.58	93.67	0.683	4.3	3.781	7.9	0.040	6.7	0.8	254	17	1589	66	4696	62

Reference materials (continued): BCR and BHVO for quality control purpuses

SPOT	238U(ppm)	232Th(ppm)	238U / 232Th	207Pb(cps)	206Pb(cps)	208Pb(cps)	202Hg(cps)	204Pb(cps)	206Pb/208Pb	207Pb/208Pb	206Pb/204Pb	2sigma(%)	207Pb/204Pb	2sigma(%)	208Pb/204Pb	2sigma(%)	207Pb/206Pb	2sigma(%)
**BCR**																		
BCR 1 - 1	1.7	6.3	3.8	381647	434262	381647	39965	24116	0.484	0.404	18.80	0.6	15.67	0.6	38.82	0.6	0.83	0.2
BCR 1 - 2	1.7	6.3	3.8	375871	427264	375871	40049	23809	0.484	0.404	18.76	0.5	15.65	0.6	38.78	0.6	0.83	0.2
BCR 1 - 3	1.7	6.4	3.8	384113	436721	384113	40014	24138	0.484	0.404	18.71	0.5	15.60	0.6	38.65	0.6	0.83	0.2
BCR 1 - 4	1.6	6.0	3.7	369287	419699	369287	40322	23043	0.483	0.404	18.66	0.7	15.58	0.7	38.60	0.7	0.83	0.3
BCR 1 - 5	1.6	5.9	3.7	361331	410939	361331	40271	22515	0.484	0.404	18.77	0.6	15.65	0.6	38.77	0.6	0.83	0.2
BCR 1 - 6	1.7	6.3	3.7	384761	437344	384761	39895	24098	0.484	0.404	18.71	0.5	15.61	0.6	38.68	0.6	0.83	0.2
BCR 1 - 7	1.6	6.0	3.6	373884	425002	373884	39877	23066	0.484	0.404	18.69	0.6	15.59	0.6	38.63	0.6	0.83	0.2
BCR 1 - 8	1.7	6.3	3.7	390860	444083	390860	34740	23999	0.484	0.404	18.79	0.5	15.68	0.6	38.85	0.6	0.83	0.2
BCR 1 - 9	1.7	6.2	3.7	387131	440372	387131	35134	23494	0.484	0.404	18.83	0.5	15.70	0.6	38.90	0.6	0.83	0.2
BCR 1 - 10	1.6	6.0	3.7	376140	427453	376140	34451	22892	0.484	0.404	18.78	0.5	15.67	0.6	38.82	0.6	0.83	0.2
BCR 1 - 11	1.7	6.3	3.7	389961	442645	389961	32088	23642	0.483	0.404	18.80	0.5	15.70	0.6	38.90	0.6	0.84	0.2
BCR 1 - 12	1.7	6.4	3.8	391988	445099	391988	31807	23900	0.483	0.404	18.73	0.5	15.64	0.6	38.75	0.6	0.84	0.2
BCR 1 - 13	1.5	5.9	3.8	354995	402791	354995	31693	21352	0.483	0.404	18.76	0.9	15.68	0.9	38.84	0.9	0.84	0.3
BCR 1 - 14	1.7	6.6	3.8	399420	453274	399420	31484	24282	0.483	0.404	18.77	0.5	15.68	0.6	38.86	0.6	0.84	0.2
									**Average (2%)**		**18.75**	**0.6**	**15.65**	**0.6**	**38.78**	**0.6**	**0.83**	**0.2**
									**Woodhead and Hergt 2000**		**18.81**	**0.1**	**15.64**	**0.1**	**38.70**	**0.1**	**0.83**	**0.2**

SPOT	238U(ppm)	232Th(ppm)	238U / 232Th	207Pb(cps)	206Pb(cps)	208Pb(cps)	202Hg(cps)	204Pb(cps)	206Pb/208Pb	207Pb/208Pb	206Pb/204Pb	2sigma(%)	207Pb/204Pb	2sigma(%)	208Pb/204Pb	2sigma(%)	207Pb/206Pb	2sigma(%)
**BHVO**																		
BHVO - 2 -1	0.4	1.2	2.9	64735	73746	64735	40065	4611	0.484	0.404	18.69	1.0	15.60	1.0	38.65	1.0	0.83	0.3
BHVO - 2 -2	0.4	1.2	3.0	64844	73682	64844	39521	4435	0.484	0.404	18.76	1.3	15.65	1.3	38.77	1.3	0.83	0.3
BHVO - 2 -3	0.4	1.2	2.9	63580	72430	63580	39290	4517	0.484	0.404	18.63	0.9	15.54	1.0	38.49	1.0	0.83	0.3
BHVO - 2 -4	0.4	1.2	2.9	63228	72243	63228	39279	4473	0.485	0.404	18.68	1.0	15.53	1.0	38.48	1.0	0.83	0.3
BHVO - 2 -5	0.4	1.2	2.9	65053	74233	65053	39063	4631	0.485	0.404	18.71	1.1	15.58	1.2	38.60	1.2	0.83	0.3
BHVO - 2 -6	0.4	1.2	2.9	62138	70610	62138	39125	4441	0.483	0.404	18.65	2.3	15.59	2.2	38.64	2.2	0.84	0.4
BHVO - 2 -7	0.4	1.2	2.9	62036	70880	62036	38907	4389	0.485	0.404	18.65	1.0	15.51	1.0	38.43	1.0	0.83	0.3
BHVO - 2 -8	0.4	1.2	2.9	62297	73744	62297	38747	4512	0.484	0.404	18.64	1.2	15.54	1.2	38.52	1.2	0.83	0.3
									**Average (2%)**		**18.68**	**1.2**	**15.57**	**1.2**	**38.57**	**1.2**	**0.83**	**0.3**
									**Woodhead and Hergt 2000**		**18.64**	**0.1**	**15.54**	**0.1**	**38.23**	**0.1**	**0.83**	**0.2**

Table S2 - In-situ LA-ICPMS Sr isotope data

sample /spot number	88Sr	$^{87}Sr/^{86}Sr_{raw}$	$^{87}Sr/^{86}Sr_{Kr}$	$^{87}Sr/^{86}Sr_{REE}$	$^{87}Sr/^{86}Sr_{Ca}$	$^{87}Sr/^{86}Sr_{Rb}$	**$^{87}Sr/^{86}Sr_{i}$**	**± 2 S.E.**	$^{84}Sr/^{86}Sr_{raw}$	$^{84}Sr/^{86}Sr_{Kr}$	$^{84}Sr/^{86}Sr_{Ree}$	$^{84}Sr/^{86}Sr$	± 2 S.E.	$^{84}Sr/^{88}Sr$	± 2 S.E.	$^{87}Rb/^{86}Sr$	± 2 S.E.
SMB1																	
031Sr	3.59	0.70697864	0.707255716	0.707221674	0.707221674	0.707225064	**0.707225**	**0.000029**	0.058215882	0.056641716	0.056574411	0.056574	3.47E-05	0.006752	4.14E-06	-1.2E-05	1.32E-05
032Sr	1.49	0.706749288	0.707376769	0.70731636	0.70731636	0.707274566	**0.707275**	**0.000053**	0.060452617	0.056693768	0.056582924	0.056592	6.44E-05	0.006754	7.68E-06	5E-05	2.99E-05
037Sr	2.04	0.706772197	0.707229989	0.7071758	0.7071758	0.707205008	**0.707205**	**0.000054**	0.059310755	0.056583268	0.056483732	0.056486	7.44E-05	0.006742	8.89E-06	-3.3E-05	2.22E-05
038Sr	2.82	0.706886864	0.707244873	0.70720965	0.70720965	0.707204742	**0.707205**	**0.000036**	0.058516982	0.056627562	0.056572226	0.056571	4.2E-05	0.006752	5.01E-06	8.72E-06	1.6E-05
039Sr	4.24	0.706974257	0.707225496	0.707191615	0.707191615	0.707206523	**0.707207**	**0.000032**	0.057798253	0.056572057	0.056537746	0.056538	2.9E-05	0.006748	3.46E-06	-4.2E-06	1.04E-05
040Sr	2.42	0.706857557	0.707217319	0.707186731	0.707186731	0.707202889	**0.707203**	**0.000039**	0.058777412	0.056564245	0.056504811	0.056499	4.61E-05	0.006743	5.5E-06	-1.8E-05	1.59E-05
042Sr	2.55	0.706797089	0.707231969	0.707170809	0.707170809	0.707220923	**0.707221**	**0.000040**	0.058687959	0.056526181	0.056467215	0.056469	4.49E-05	0.00674	5.35E-06	-4.1E-05	1.47E-05
044Sr	1.76	0.706673888	0.707244417	0.70720161	0.70720161	0.707222318	**0.707222**	**0.000059**	0.059653871	0.056584768	0.056539613	0.056538	6.79E-05	0.006748	8.1E-06	-1.4E-05	1.94E-05
086Sr	0.79	0.71989899	0.721327164	0.721129085	0.721129085	0.707266238	**0.707266**	**0.000107**	0.063532011	0.056796296	0.056614922	0.056593	0.00013	0.006754	1.55E-05	0.014122	0.001626
087Sr	0.97	0.715328973	0.716250483	0.716203062	0.716203062	0.707397897	**0.707398**	**0.000084**	0.062138195	0.056798399	0.056578969	0.056576	0.00012	0.006752	1.43E-05	0.008864	0.001268
089Sr	0.84	0.743712862	0.744787701	0.744646845	0.744646845	0.70726617	**0.707266**	**0.000080**	0.063024846	0.056736028	0.056586701	0.056589	0.00012	0.006754	1.43E-05	0.036779	0.001793
090Sr	0.93	0.72311673	0.724236653	0.724056815	0.724056815	0.707237694	**0.707237**	**0.000092**	0.062419614	0.056617199	0.056496425	0.056509	0.000109	0.006744	1.3E-05	0.01644	0.001113
092Sr	0.91	0.714989043	0.716053507	0.715925275	0.715925275	0.707221363	**0.707221**	**0.000087**	0.06255508	0.056587365	0.056398358	0.056409	0.000124	0.006732	1.48E-05	0.008981	0.001612
094Sr	0.84	0.717290938	0.718598543	0.718497386	0.718497386	0.707249941	**0.707250**	**0.000092**	0.062887812	0.056846209	0.056611708	0.056618	0.000132	0.006757	1.58E-05	0.011199	0.0008
096Sr	1.84	0.706763261	0.707295088	0.707217307	0.707217307	0.707228142	**0.707228**	**0.000052**	0.059368881	0.056592035	0.056543227	0.056549	5.58E-05	0.006749	6.66E-06	2.41E-06	1.64E-05
098Sr	1.86	0.706832921	0.707462358	0.707432444	0.707432444	0.70739883	**0.707399**	**0.000052**	0.05939111	0.056432954	0.056360792	0.056371	5.65E-05	0.006728	6.74E-06	3.55E-05	2.39E-05
100Sr	1.16	0.706632574	0.707542408	0.707427792	0.707427792	0.70723247	**0.707232**	**0.000098**	0.06112201	0.05678168	0.056692755	0.056693	8.76E-05	0.006766	1.05E-05	0.000192	7.06E-05
101Sr	1.90	0.706737945	0.707223788	0.707155504	0.707155504	0.707203158	**0.707203**	**0.000051**	0.05927399	0.056554729	0.056471119	0.056471	6.34E-05	0.00674	7.57E-06	-4.3E-05	2E-05
102Sr	1.07	0.706306406	0.707314549	0.707227728	0.707227728	0.707232057	**0.707232**	**0.000064**	0.061442359	0.056546524	0.056446592	0.056462	0.000103	0.006739	1.23E-05	8.25E-06	3.16E-05
103Sr	0.98	0.706328153	0.707355049	0.707231115	0.707231115	0.707249279	**0.707249**	**0.000084**	0.061871229	0.056689042	0.056583898	0.056583	0.000119	0.006753	1.41E-05	-1E-05	3.67E-05
104Sr	1.53	0.706681384	0.707332508	0.707241285	0.707241285	0.707238685	**0.707239**	**0.000069**	0.06004539	0.056802046	0.056685769	0.056685	7.3E-05	0.006765	8.72E-06	-5.8E-06	3.08E-05
105Sr	1.21	0.706523	0.707414943	0.707350467	0.707350467	0.707225939	**0.707226**	**0.000059**	0.060872372	0.056706002	0.056590147	0.05659	9.45E-05	0.006754	1.13E-05	0.000142	5.37E-05
106Sr	2.41	0.706865714	0.707289941	0.707241889	0.707241889	0.707211392	**0.707211**	**0.000040**	0.058666335	0.056554879	0.056489687	0.05649	4.51E-05	0.006742	5.38E-06	3.62E-05	1.74E-05
108Sr	3.01	0.707008861	0.707349947	0.707330061	0.707330061	0.70721207	**0.707212**	**0.000031**	0.0581866	0.056513209	0.056481391	0.056481	4.14E-05	0.006741	4.94E-06	0.000108	1.92E-05
109Sr	2.48	0.706866745	0.707276288	0.707215829	0.707215829	0.707206373	**0.707206**	**0.000033**	0.05864728	0.056634413	0.056581345	0.056587	5.03E-05	0.006754	6.01E-06	2.51E-06	1.54E-05
110Sr	2.83	0.706939094	0.707287722	0.707259723	0.707259723	0.707243292	**0.707243**	**0.000029**	0.058262637	0.056483954	0.056436299	0.056435	3.96E-05	0.006736	4.73E-06	1.16E-05	1.45E-05
116Sr	3.05	0.706873663	0.707224538	0.707202394	0.707202394	0.707207299	**0.707207**	**0.000039**	0.058236402	0.056567274	0.056513512	0.05651	5.41E-05	0.006744	6.46E-06	-1.5E-05	1.27E-05
117Sr	2.55	0.706910908	0.70729685	0.707244389	0.707244389	0.707229274	**0.707229**	**0.000034**	0.05854338	0.056604242	0.056526411	0.056526	4.55E-05	0.006745	5.44E-06	1.5E-05	1.48E-05
119Sr	3.15	0.706972073	0.707296021	0.707257853	0.707257853	0.707215778	**0.707216**	**0.000034**	0.058117756	0.056544556	0.056516831	0.056518	3.61E-05	0.006744	4.31E-06	4.21E-05	1.66E-05
120Sr	2.78	0.707066187	0.707447044	0.707397934	0.707397934	0.707254933	**0.707255**	**0.000039**	0.058327112	0.056567554	0.056496697	0.056497	4.13E-05	0.006742	4.93E-06	0.00014	3.2E-05
121Sr	3.97	0.70701622	0.707249258	0.707231931	0.707231931	0.707238542	**0.707239**	**0.000020**	0.05776439	0.05654968	0.056510204	0.056504	3.68E-05	0.006743	4.39E-06	-2.6E-06	8.73E-06
122Sr	4.42	0.707066699	0.707288323	0.707264031	0.707264031	0.707249565	**0.707250**	**0.000026**	0.057610797	0.056512949	0.056489314	0.056489	2.26E-05	0.006741	2.69E-06	7.92E-06	8.43E-06
124Sr	3.34	0.70693028	0.707235205	0.707207684	0.707207684	0.707225863	**0.707226**	**0.000028**	0.058023427	0.056476119	0.056396904	0.056393	3.78E-05	0.006732	4.52E-06	-1.1E-05	1.54E-05
125Sr	3.43	0.707101699	0.707381037	0.70733519	0.70733519	0.707233562	**0.707234**	**0.000036**	0.058017072	0.056586787	0.056525093	0.05652	6.39E-05	0.006747	7.63E-06	0.000101	2.38E-05
127Sr	3.33	0.707049238	0.707317009	0.707290898	0.707290898	0.707202101	**0.707202**	**0.000034**	0.058002035	0.056499309	0.056431272	0.056433	5.2E-05	0.006737	6.2E-06	9.68E-05	3.5E-05
128Sr	3.59	0.707095672	0.707310477	0.707291933	0.707291933	0.707210231	**0.707210**	**0.000028**	0.057934404	0.056593989	0.056558372	0.05656	3.56E-05	0.006752	4.25E-06	8.45E-05	2.31E-05
129Sr	4.45	0.707020015	0.707280104	0.707229465	0.707229465	0.707215451	**0.707215**	**0.000044**	0.057749487	0.056592336	0.056519434	0.056519	4.27E-05	0.006747	5.1E-06	1.39E-05	1.23E-05
130Sr	3.75	0.706960582	0.707248679	0.707207633	0.707207633	0.707202032	**0.707202**	**0.000040**	0.057859707	0.056550903	0.05651086	0.056513	3.48E-05	0.006747	4.16E-06	2.6E-07	9.33E-06
RP1 - stromatolite columns																	
171Sr	3.30	0.708565854	0.708849157	0.708809971	0.708809971	0.707280968	**0.707281**	**0.000034**	0.057967296	0.056544205	0.056522707	0.056522	3.28E-05	0.006746	3.91E-06	0.001509	0.000263
172Sr	3.39	0.708459351	0.708740149	0.708663071	0.708663071	0.707262226	**0.707262**	**0.000032**	0.057966513	0.056551398	0.056543928	0.056547	3.36E-05	0.006749	4.01E-06	0.00136	0.000227
173Sr	3.35	0.708515228	0.708798587	0.70872419	0.70872419	0.707267016	**0.707267**	**0.000035**	0.057977521	0.056548332	0.056531851	0.056536	3.84E-05	0.006748	4.59E-06	0.001411	0.000243
174Sr	4.01	0.707711017	0.707920277	0.707877731	0.707877731	0.707274317	**0.707274**	**0.000034**	0.057723017	0.056535304	0.056508575	0.05651	2.94E-05	0.006745	3.51E-06	0.000585	0.000113
175Sr	3.63	0.707103877	0.707358987	0.707336521	0.707336521	0.707273237	**0.707273**	**0.000024**	0.057855581	0.056541921	0.056526848	0.056521	3.76E-05	0.006746	4.49E-06	6.22E-05	1.58E-05

176Sr	2.37	0.707109336	0.707421761	0.707353587	0.707353587	0.70726087	**0.707261**	**0.000046**	0.058610388	0.056545349	0.056504743	0.056502	4.4E-05	0.006743	5.25E-06	8.37E-05	2.36E-05
177Sr	3.11	0.710058832	0.71039951	0.710357917	0.710357917	0.70742771	**0.707428**	**0.000032**	0.058068805	0.056566903	0.056526626	0.056523	3.56E-05	0.006746	4.25E-06	0.002897	0.000652
178Sr	3.08	0.707403743	0.707711486	0.707674729	0.707674729	0.707268485	**0.707268**	**0.000032**	0.058085682	0.056525642	0.056496707	0.056493	4.87E-05	0.006742	5.81E-06	0.000399	7.9E-05
RP2 - Black micrite matrix																	
163Sr	1.12	0.730775994	0.731519013	0.731381662	0.731381662	0.707786645	**0.708186**	**0.000074**	0.060873328	0.056569921	0.056438691	0.05643	0.000104	0.006735	1.24E-05	0.023229	0.001311
165Sr	1.37	0.73799376	0.738725659	0.738626133	0.738626133	0.707956025	**0.708156**	**0.000057**	0.060088409	0.056691718	0.056619198	0.056607	7.26E-05	0.006756	8.67E-06	0.03027	0.001449
166Sr	1.30	0.726875436	0.727614975	0.727566269	0.727566269	0.707921616	**0.708121**	**0.000051**	0.060252478	0.056579804	0.056450552	0.056451	0.000108	0.006737	1.29E-05	0.019216	0.000969
167Sr	1.10	0.736165862	0.737066064	0.736958099	0.736958099	0.707917933	**0.708118**	**0.000061**	0.06092845	0.056614207	0.056422902	0.056425	9.8E-05	0.006734	1.17E-05	0.029061	0.00153
TATIB-1																	
071Sr	1.59	0.727509801	0.728253318	0.728223134	0.728223134	0.708297436	**0.708197**	**0.000049**	0.059989017	0.056662922	0.05657801	0.056582	7.22E-05	0.006753	8.62E-06	0.019966	0.000825
072Sr	1.89	0.71901962	0.719602483	0.719551693	0.719551693	0.707978524	**0.708078**	**0.000038**	0.059488688	0.056529145	0.056453489	0.056453	5.71E-05	0.006738	6.81E-06	0.011472	0.000659
073Sr	1.89	0.726062982	0.726659289	0.726611058	0.726611058	0.708073379	**0.708073**	**0.000041**	0.059506136	0.056581099	0.056526182	0.056526	5.88E-05	0.006746	7.02E-06	0.018395	0.000666
074Sr	1.79	0.730782548	0.731453599	0.731364338	0.731364338	0.708058587	**0.708058**	**0.000045**	0.059611549	0.056539552	0.056454402	0.056458	6.74E-05	0.006738	8.04E-06	0.022914	0.002257
075Sr	1.87	0.722791697	0.723384656	0.723327788	0.723327788	0.708039053	**0.708039**	**0.000041**	0.059465812	0.056655532	0.056579318	0.056576	6.03E-05	0.006752	7.2E-06	0.015035	0.000772
076Sr	1.58	0.725470173	0.72614916	0.726052449	0.726052449	0.708017117	**0.708017**	**0.000048**	0.060055008	0.056640509	0.056568832	0.056567	8.54E-05	0.006751	1.02E-05	0.017945	0.000859
077Sr	1.51	0.733277562	0.734084569	0.733934839	0.733934839	0.708048493	**0.708048**	**0.000089**	0.060264187	0.056642887	0.05658789	0.0566	7.64E-05	0.006755	9.11E-06	0.026355	0.004233
079Sr	1.44	0.722138156	0.722871652	0.722832761	0.722832761	0.70807289	**0.708073**	**0.000055**	0.060270842	0.056419596	0.056333335	0.056332	8.3E-05	0.006723	9.91E-06	0.014613	0.000964
080Sr	1.41	0.725012539	0.725862613	0.725778457	0.725778457	0.708074875	**0.708075**	**0.000069**	0.060368974	0.056530688	0.056458655	0.05645	8.47E-05	0.006737	1.01E-05	0.01764	0.001377
TATIB-2																	
141Sr	1.38	0.722082114	0.722974	0.722900823	0.722900823	0.708536388	**0.708536**	**0.000052**	0.06009414	0.056682206	0.056548668	0.056558	9.11E-05	0.006752	1.09E-05	0.01428	0.001637
143Sr	1.44	0.732237127	0.733022547	0.732960428	0.732960428	0.708397193	**0.708497**	**0.000055**	0.060000512	0.056573573	0.056505643	0.056508	7.58E-05	0.006746	9.05E-06	0.02463	0.001613
144Sr	1.28	0.73616217	0.736929779	0.736767116	0.736767116	0.708444726	**0.708444**	**0.000061**	0.060353387	0.056702932	0.056607968	0.056609	8.33E-05	0.006758	9.95E-06	0.027946	0.001518
145Sr	1.28	0.724449959	0.725177647	0.725087444	0.725087444	0.708674953	**0.708475**	**0.000059**	0.060419567	0.056710349	0.056671777	0.056673	7.53E-05	0.006766	8.99E-06	0.016799	0.001909
146Sr	1.49	0.712536171	0.713212457	0.713062605	0.713062605	0.70795021	**0.708450**	**0.000067**	0.059868732	0.056590009	0.056450126	0.056451	8.17E-05	0.006739	9.76E-06	0.005233	0.000771
147Sr	1.41	0.726622833	0.727329148	0.727268076	0.727268076	0.708065261	**0.708465**	**0.000057**	0.059938458	0.056632252	0.056542347	0.056534	8.61E-05	0.006749	1.03E-05	0.018983	0.000795
148Sr	1.43	0.716808293	0.717481316	0.717363827	0.717363827	0.708482711	**0.708483**	**0.000074**	0.060123665	0.056762337	0.056654208	0.056656	8.29E-05	0.006764	9.9E-06	0.008833	0.000264
149Sr	1.39	0.724934874	0.7256093	0.72556691	0.72556691	0.708092288	**0.708492**	**0.000051**	0.060050723	0.056553193	0.056425868	0.056423	7.3E-05	0.006736	8.72E-06	0.017667	0.002235
150Sr	1.41	0.721330248	0.722067384	0.722008412	0.722008412	0.708171614	**0.708471**	**0.000053**	0.06003871	0.056643455	0.056505258	0.056505	8.96E-05	0.006746	1.07E-05	0.013627	0.0009
TATIA ARAGONITE FANS																	
056Sr	3.35	0.707242782	0.707558817	0.707503385	0.707503385	0.707512792	**0.707513**	**0.000032**	0.058273421	0.056467018	0.056408532	0.056409	3.35E-05	0.006732	4E-06	-9.4E-06	1.08E-05
059Sr	3.69	0.707367323	0.707651384	0.707618072	0.707618072	0.707627826	**0.707528**	**0.000033**	0.058027744	0.05654842	0.056513305	0.056512	3.03E-05	0.006745	3.61E-06	-6.6E-06	9.83E-06
061Sr	3.97	0.707276063	0.707560876	0.707510651	0.707510651	0.707517944	**0.707518**	**0.000028**	0.057894131	0.056496757	0.056448785	0.056449	3.26E-05	0.006737	3.9E-06	-3.2E-06	1.15E-05
063Sr	4.36	0.707297236	0.707556309	0.707526971	0.707526971	0.707534117	**0.707534**	**0.000027**	0.057777571	0.056502404	0.056462956	0.05646	3.24E-05	0.006739	3.86E-06	-6.8E-06	8.07E-06
068Sr	3.20	0.707299448	0.707645316	0.70758116	0.70758116	0.707583044	**0.707523**	**0.000037**	0.058219477	0.056563064	0.056496418	0.056503	4.76E-05	0.006744	5.68E-06	-2.5E-07	1.45E-05
069Sr	3.69	0.707303594	0.707587685	0.707560902	0.707560902	0.707568806	**0.707539**	**0.000031**	0.057996995	0.056636324	0.056616109	0.056617	2.87E-05	0.006757	3.42E-06	-2.3E-06	1.05E-05
070Sr	4.00	0.707376463	0.707658856	0.707612271	0.707612271	0.707577442	**0.707537**	**0.000037**	0.05785783	0.056504148	0.056459465	0.056458	3.52E-05	0.006738	4.2E-06	3.39E-05	2.92E-05
131Sr	4.05	0.707346753	0.707582966	0.707535926	0.707535926	0.707545438	**0.707545**	**0.000022**	0.057735559	0.056510355	0.056469088	0.056468	2.91E-05	0.006741	3.47E-06	-1.1E-05	9.61E-06
132Sr	2.95	0.70726294	0.707563229	0.707519933	0.707519933	0.707506936	**0.707507**	**0.000029**	0.058184196	0.056544353	0.056515467	0.05651	4.87E-05	0.006746	5.81E-06	1.61E-05	1.63E-05
133Sr	3.26	0.707294961	0.707599408	0.707513546	0.707513546	0.707528745	**0.707529**	**0.000026**	0.057983638	0.056520653	0.05645588	0.056462	3.48E-05	0.00674	4.15E-06	-1.5E-05	1.26E-05
134Sr	3.50	0.707322381	0.707579077	0.707535976	0.707535976	0.707512496	**0.707512**	**0.000032**	0.057920711	0.056545166	0.056518946	0.056519	3.5E-05	0.006747	4.18E-06	1.69E-05	1.05E-05
136Sr	3.65	0.707293404	0.707565109	0.707532547	0.707532547	0.707537333	**0.707537**	**0.000025**	0.057854305	0.056513815	0.056465728	0.056465	2.66E-05	0.006741	3.18E-06	-2.7E-06	1.04E-05
137Sr	3.78	0.70728958	0.707534612	0.707501767	0.707501767	0.707500645	**0.707501**	**0.000028**	0.057784113	0.056525582	0.056498499	0.056503	2.39E-05	0.006745	2.86E-06	4.15E-06	1.1E-05
139Sr	3.43	0.707290365	0.707581568	0.707507666	0.707507666	0.707523236	**0.707523**	**0.000029**	0.057938206	0.056507038	0.056465402	0.05647	4.16E-05	0.006741	4.96E-06	-9.4E-06	1.22E-05
140Sr	3.44	0.70729143	0.707561581	0.70751499	0.70751499	0.707510609	**0.707511**	**0.000029**	0.057990763	0.056629624	0.056584905	0.056585	2.77E-05	0.006755	3.31E-06	1.72E-06	1.1E-05

Standards data

Sample	^{88}Sr	$^{87}Sr/^{86}Sr_{raw}$	$^{87}Sr/^{86}Sr_{Kr}$	$^{87}Sr/^{86}Sr_{REE}$	$^{87}Sr/^{86}Sr_{Ca}$	$^{87}Sr/^{86}Sr_{Rb}$	**87Sr/86Sri**	**± 2 S.E.**	$^{84}Sr/^{86}Sr_{raw}$	$^{84}Sr/^{86}Sr_{Kr}$	$^{84}Sr/^{86}Sr_{Ree}$	$^{84}Sr/^{86}Sr$	± 2 S.E.	$^{84}Sr/^{88}Sr$	± 2 S.E.	$^{87}Rb/^{86}Sr$	± 2 S.E.
MIR PLAGIOCLASE																	
083Sr.dat	1.24	0.704715961	0.705655958	0.705598635	0.705598635	0.703169879	**0.703170**	**0.000053**	0.060665304	0.056226142	0.056123555	0.056124	9.28E-05	0.006698	1.11E-05	0.002407	3.55E-05
050Sr.dat	1.55	0.705006106	0.705497909	0.70545725	0.70545725	0.703098757	**0.703099**	**0.000050**	0.059701981	0.056334556	0.056330793	0.056328	0.000117	0.006723	1.4E-05	0.002337	4.19E-05
113Sr.dat	1.20	0.70475718	0.705509456	0.705419438	0.705419438	0.703074302	**0.703074**	**0.000039**	0.060455213	0.056232653	0.056123717	0.056116	9.65E-05	0.006697	1.15E-05	0.002323	2.92E-05
001Sr.dat	1.83	0.705047277	0.705534503	0.705482148	0.705482148	0.703070354	**0.703070**	**0.000034**	0.059998149	0.056423388	0.056347972	0.056357	5.99E-05	0.006726	7.14E-06	0.002393	2.32E-05
007Sr.dat	1.48	0.704944796	0.705585173	0.705500421	0.705500421	0.703068733	**0.703069**	**0.000053**	0.060553728	0.056638081	0.056555629	0.056564	6.86E-05	0.006751	8.19E-06	0.002403	2.2E-05
084Sr.dat	1.16	0.70453483	0.705527359	0.705445053	0.705445053	0.703064353	**0.703064**	**0.000057**	0.060887145	0.056173839	0.056058673	0.056069	9.89E-05	0.006692	1.18E-05	0.002359	3.01E-05
154Sr.dat	1.19	0.704781432	0.70557032	0.705449917	0.705449917	0.703063488	**0.703063**	**0.000083**	0.060313528	0.056132004	0.055968466	0.055968	0.000137	0.00668	1.64E-05	0.002355	2.68E-05
182Sr.dat	-0.59	0.576426126	0.70557248	0.705852856	0.705852856	0.703062484	**0.703062**	**0.000180**	0.567161115	0.06668686	0.067277064	0.067071	0.001545	0.008005	0.000184	0.002746	0.000105
114Sr.dat	1.31	0.704877439	0.70558474	0.70551482	0.70551482	0.703055462	**0.703055**	**0.000072**	0.060134898	0.056271742	0.056219412	0.056222	8.25E-05	0.00671	9.85E-06	0.002418	2.97E-05
004Sr.dat	1.76	0.7050061	0.70557693	0.705490922	0.705490922	0.703050697	**0.703051**	**0.000048**	0.059869486	0.056348967	0.056272117	0.056282	7.02E-05	0.006717	8.38E-06	0.002416	1.91E-05
115Sr.dat	1.29	0.70471937	0.705531288	0.7054421	0.7054421	0.703049659	**0.703050**	**0.000060**	0.060269329	0.056373625	0.056232531	0.05621	9.36E-05	0.006709	1.12E-05	0.002369	3.14E-05
081Sr.dat	1.26	0.704823177	0.705571285	0.705500503	0.705500503	0.703036022	**0.703036**	**0.000095**	0.060694475	0.056336144	0.056175199	0.056164	9.67E-05	0.006703	1.15E-05	0.002416	3.5E-05
082Sr.dat	1.24	0.704705808	0.705567652	0.705471894	0.705471894	0.703033735	**0.703034**	**0.000047**	0.060775416	0.056301456	0.056160558	0.056154	0.000115	0.006702	1.38E-05	0.002414	3.37E-05
005Sr.dat	1.60	0.704849019	0.705592132	0.705431709	0.705431709	0.703031856	**0.703032**	**0.000058**	0.060331029	0.056714913	0.056486099	0.056492	9.86E-05	0.006744	1.18E-05	0.002395	2.73E-05
185Sr.dat	1.10	0.704772459	0.705559396	0.705505404	0.705505404	0.703031122	**0.703031**	**0.000063**	0.060646633	0.056229592	0.055957554	0.05594	9.78E-05	0.006676	1.17E-05	0.002441	2.92E-05
085Sr.dat	1.12	0.704665222	0.705626054	0.705521573	0.705521573	0.703028743	**0.703029**	**0.000083**	0.061035997	0.056225681	0.056008429	0.056019	0.000146	0.006686	1.74E-05	0.002446	4.07E-05
001Sr.dat	1.79	0.705034691	0.705532357	0.705437352	0.705437352	0.703027221	**0.703027**	**0.000045**	0.060095726	0.056390812	0.056297764	0.056312	7.84E-05	0.006722	9.36E-06	0.002393	3.08E-05
MAD APATITE																	
017Sr.dat	1.64	0.711240995	0.711829275	0.711658614	0.711658614	0.711709749	**0.711709**	**0.000051**	0.060931468	0.057500326	0.056595663	0.05659	8.49E-05	0.006756	1.01E-05	-6.7E-05	2.2E-05
016Sr.dat	1.63	0.71123753	0.711873054	0.711603691	0.711603691	0.711709424	**0.711709**	**0.000083**	0.06102652	0.057426739	0.056401516	0.056414	9.58E-05	0.006735	1.14E-05	-0.00012	2.79E-05
017Sr.dat	1.64	0.711240995	0.711829275	0.711658614	0.711658614	0.711709749	**0.711710**	**0.000051**	0.060931468	0.057500326	0.056595663	0.05659	8.49E-05	0.006756	1.01E-05	-6.7E-05	2.2E-05
018Sr.dat	1.63	0.711308471	0.711792697	0.711641601	0.711641601	0.711700871	**0.711701**	**0.000067**	0.060915326	0.057348192	0.056439731	0.05644	9.68E-05	0.006738	1.16E-05	-5.4E-05	2.96E-05
019Sr.dat	1.63	0.711225163	0.711794449	0.711620238	0.711620238	0.711718281	**0.711718**	**0.000050**	0.060907501	0.057411599	0.056502393	0.056502	0.000104	0.006745	1.24E-05	-9.8E-05	2.91E-05
020Sr.dat	1.65	0.711304008	0.711885067	0.711637086	0.711637086	0.711716137	**0.711716**	**0.000051**	0.060937875	0.057564158	0.056574047	0.056581	9.63E-05	0.006755	1.15E-05	-7.4E-05	2.92E-05
CORAL																	
021Sr.dat	8.14	0.709100575	0.709212416	0.709191366	0.709191366	0.709142595	**0.709143**	**0.000019**	0.057232439	0.056519499	0.056503056	0.056506	1.68E-05	0.006744	2E-06	5.36E-05	5.34E-06
022Sr.dat	8.12	0.709114556	0.709230599	0.709215858	0.709215858	0.709136095	**0.709136**	**0.000015**	0.057234657	0.056534999	0.056522736	0.056522	1.69E-05	0.006746	2.02E-06	7.55E-05	7.37E-06
023Sr.dat	8.44	0.709142151	0.70926486	0.709246251	0.709246251	0.709174857	**0.709175**	**0.000017**	0.057210298	0.056559604	0.056540891	0.056538	1.52E-05	0.006748	1.82E-06	7.23E-05	6.86E-06
024Sr.dat	5.53	0.709019062	0.709210131	0.709182127	0.709182127	0.709128298	**0.709128**	**0.000033**	0.057521427	0.056539198	0.056525046	0.056529	2.75E-05	0.006747	3.28E-06	4.51E-05	1.13E-05
025Sr.dat	5.00	0.709046529	0.709269528	0.709248475	0.709248475	0.709169462	**0.709169**	**0.000022**	0.05767215	0.056521611	0.056508066	0.056511	2.26E-05	0.006745	2.7E-06	8.08E-05	9.25E-06
011Sr.dat	0.29	0.705992948	0.709460173	0.708981685	0.708981685	0.709101555	**0.709102**	**0.000256**	0.080074371	0.060353853	0.057858132	0.05793	0.000651	0.006914	7.77E-05	-0.00016	0.000144
051Sr.dat	7.67	0.709056463	0.709195659	0.709183473	0.709183473	0.709142218	**0.709142**	**0.000020**	0.057255664	0.056513508	0.056504724	0.056506	1.8E-05	0.006744	2.14E-06	4.32E-05	6.47E-06
052Sr.dat	6.65	0.709056882	0.709219438	0.709205861	0.709205861	0.709154598	**0.709155**	**0.000022**	0.057422382	0.056617458	0.056576454	0.056572	5.09E-05	0.006752	6.08E-06	4.56E-05	6.51E-06
053Sr.dat	6.70	0.709121191	0.709262322	0.709240292	0.709240292	0.709152021	**0.709152**	**0.000026**	0.057322929	0.056515279	0.05650409	0.056508	2.3E-05	0.006744	2.75E-06	8.69E-05	5.88E-06
054Sr.dat	6.99	0.709100293	0.709239659	0.709220999	0.709220999	0.70914741	**0.709147**	**0.000018**	0.057350011	0.056535567	0.056500134	0.0565	2.45E-05	0.006743	2.92E-06	7.19E-05	7.23E-06
055Sr.dat	7.28	0.709105978	0.709262187	0.709239643	0.709239643	0.709154036	**0.709154**	**0.000017**	0.057323986	0.056505252	0.056477711	0.05648	2.07E-05	0.006741	2.47E-06	8.88E-05	5.44E-06
156Sr.dat	5.58	0.70905454	0.709225279	0.709199348	0.709199348	0.709138669	**0.709139**	**0.000017**	0.057385902	0.056538099	0.056506245	0.056508	2.15E-05	0.006744	2.56E-06	6.06E-05	6E-06
157Sr.dat	5.53	0.709103219	0.709269279	0.709251337	0.709251337	0.709165106	**0.709165**	**0.000019**	0.05739939	0.056532002	0.056518842	0.056521	2.16E-05	0.006746	2.57E-06	8.75E-05	1.02E-05
158Sr.dat	5.63	0.709093156	0.709251341	0.709212555	0.709212555	0.709154122	**0.709154**	**0.000020**	0.057336341	0.056456205	0.05641786	0.05642	1.91E-05	0.006734	2.28E-06	5.81E-05	6.3E-06
159Sr.dat	4.57	0.709029179	0.70922492	0.709191923	0.709191923	0.709116554	**0.709117**	**0.000031**	0.057503618	0.056498796	0.056487092	0.056491	2.57E-05	0.006742	3.07E-06	6.8E-05	1.14E-05
160Sr.dat	5.61	0.709125776	0.709288722	0.709273432	0.709273432	0.709198725	**0.709199**	**0.000024**	0.057354401	0.05655026	0.056516536	0.056516	2.11E-05	0.006745	2.52E-06	7.16E-05	8.03E-06

186Sr.dat	4.48	0.709075575	0.709288716	0.709248784	0.709248784	0.709170672	**0.709171**	**0.000030**	0.057587726	0.056538802	0.056524915	0.056523	2.42E-05	0.006746	2.88E-06	7.66E-05	7.03E-06
187Sr.dat	5.88	0.709130113	0.70927149	0.709249725	0.709249725	0.709166015	**0.709166**	**0.000018**	0.057319283	0.056463652	0.056433963	0.056432	1.72E-05	0.006735	2.06E-06	8.29E-05	8.91E-06
BHVO BCR																	
026Sr.dat	0.30	0.775420063	0.778439147	0.778145801	0.778145801	0.703426006	**0.703425**	**0.000199**	0.076389735	0.057416156	0.056503939	0.056504	0.000382	0.006744	4.56E-05	0.073986	0.000464
027Sr.dat	0.28	0.773888854	0.777658316	0.777089529	0.777089529	0.703484428	**0.703483**	**0.000312**	0.077100523	0.056472367	0.055022414	0.055181	0.00069	0.006586	8.24E-05	0.073015	0.0003
030Sr.dat	0.26	0.773435597	0.777577174	0.777096687	0.777096687	0.703411422	**0.703410**	**0.000261**	0.079568428	0.056645845	0.055819895	0.055787	0.00047	0.006658	5.6E-05	0.072996	0.000293

**Table S3. TATI stepwise Pb leaching isotope data of aragonite fans (Tatiana quarry)**										
**sample**	**leachate**	**[Pb]**	**$^{206}Pb/^{204}Pb$**	**± 2σ⁺**	**$^{207}Pb/^{204}Pb$**	**± 2σ⁺**	**$^{208}Pb/^{204}Pb$**	**± 2σ⁺**	**r_1****	**r_2††**
Aragonite fan 1, clean										
A1 1	1N HBr	0.0015	24.9704	0.0532	16.0465	0.0380	38.2897	0.0886	0.911	0.942
A1 2	0.2% CH_3COOH	0.0023	27.6232	0.1238	16.2101	0.0731	38.3052	0.1733	0.997	0.996
A1 3	5% CH_3COOH	0.1411	28.1384	0.0239	16.2413	0.0152	38.3174	0.0401	0.976	0.927
A1 4	10% CH_3COOH	0.3127	28.3288	0.0231	16.2557	0.0147	38.1880	0.0384	0.971	0.936
A1 5	1.6N HCl	0.1385	21.2829	0.0248	15.8290	0.0196	42.0628	0.0571	0.977	0.925
A1 6	8N HCl	0.0120	53.6033	0.0800	17.7254	0.0276	39.9821	0.0662	0.981	0.943
A1 7	*aqua regia*	0.0060	28.8236	0.0733	16.2956	0.0421	39.0161	0.1025	0.991	0.982
Aragonite fan 2, clean										
A2 1	1N HBr	0.1591	27.9503	0.0803	16.2679	0.0473	38.3619	0.1127	0.994	0.990
A2 2	0.5% CH_3COOH	0.4306	27.8921	0.0486	16.2298	0.0292	38.1616	0.0708	0.984	0.971
A2 3	10% CH_3COOH	0.2340	27.8816	0.1184	16.2269	0.0695	38.1313	0.1639	0.994	0.994
A2 4	6N HCl	0.0435	42.3805	0.0559	17.0929	0.0237	39.6281	0.0576	0.981	0.961
A2 5	6N HCl-5N HNO_3 1:1	0.0126	46.5076	0.1218	17.3291	0.0461	39.9203	0.1074	0.993	0.988
Aragonite fan, impure										
A3 1	1N HBr		27.4819	0.0421	16.1907	0.0257	38.2388	0.0626	0.986	0.976
A3 2	0.5% CH_3COOH		28.0126	0.0327	16.2428	0.0201	38.2807	0.0502	0.979	0.958
A3 3	10% CH_3COOH		27.6925	0.0623	16.2045	0.0371	38.1166	0.0888	0.992	0.985
A3 4	8N HCl		32.4440	0.0685	16.5029	0.0358	38.6926	0.0854	0.984	0.979
A3 5	*aqua regia*		46.9806	0.0620	17.3573	0.0240	39.9709	0.0576	0.981	0.970

** r_1=$^{206}Pb/^{204}Pb$ vs. $^{207}Pb/^{204}Pb$ error correlation

†† r_2=$^{206}Pb/^{204}Pb$ vs. $^{208}Pb/^{204}Pb$ error correlation

\+ Errors are two standard deviations absolute

Table S4 - Whole-rock Stable Isotope Data - Tatiana Quarry (Lagoa Santa Member)

Sample/height (m)	$\delta^{13}C_{VPDB}$‰	$\delta^{18}O_{VPDB}$‰	$^{87}Sr/^{86}Sr$	+- 2SE
TA 0 - Matrix	0.35	-11.68	-	-
TA 0 - Aragonite fan	0.84	-11.67	-	-
TA 0.5 - Matrix	0.34	-11.37	0.70834	0.00001
TA 0.5 - Aragonite fan	3.12	-12.44		
TA 1 - Matrix	0.32	-11.84	0.70804	0.00001
TA 1 - Aragonite fan	0.76	-11.8	-	-
TA 1.5 - Matrix	2.1	-6.8	-	-
TA 2 - Matrix	0.04	-9.9	0.70793	0.00001
TA 2 - Aragonite fan	0.7	-10	-	-
TA 3 - Matrix	-0.13	-8.42	0.70798	0.00001
TA 3 - Aragonite fan	0.57	-8.77	-	-
TA 3.5	0.33	-9.14	0.70806	0.00003
TA 4	-2.05	-14.25	0.70798	0.00001
TA 4.5	-0.08	-8.14	0.70775	0.00001
TA 5	-0.37	-9.7	0.70787	0.00001
TA 5.5	-0.39	-7.79	0.70804	0.00001
TA 6	-	-	0.70787	0.00001
TA 6.5	-0.33	-7.22	0.70833	0.00001
TA 7	-0.37	-7.26	0.70807	0.00001
TA 7.5	-2.46	-10.9	0.70818	0.00003
TA 8	-2.26	-12.57	0.70799	0.00003
TA 8.5	-3.31	-12.26	0.70791	0.00001

Table S5 - Trace element data (ppm) for carbonate samples of the Sete Lagoas Fm., Bambuí Group, Brazil

	Element	Mg25	Al27	Si29	P31	Ca44	Sc45	Ti47	V51	Cr52
**SMB1**	1.sSMPABC008	2839.70	671.18	2374.22	<-NaN	400232.75	0.16	49.06	1.16	10.77
	1.sSMPABC009	3260.62	2797.96	5738.28	<-NaN	400232.72	0.65	100.25	3.74	13.85
**SMB2**	1.sSMPABC046	743.08	30.92	576.24	<-NaN	400232.72	0.17	5.68	0.09	8.78
	1.sSMPABC051	701.45	211.28	943.73	<-NaN	400232.69	0.18	17.09	0.30	9.44
	1.sSMPABC053	439.04	7.17	583.55	<-NaN	400232.72	0.10	<2.70	0.25	8.27
	1.sSMPABC059	1204.23	8.13	587.58	<-NaN	400232.69	0.20	<2.81	0.04	8.30
	1.sSMPABC071	681.41	56.66	798.36	<-NaN	400232.69	0.11	<3.07	0.44	10.92
	1.sSMPABC057	841.44	8.17	479.55	<-NaN	400232.72	0.15	4.20	0.02	9.05
	1.sSMPABC072	1025.15	75.50	725.64	<-NaN	400232.66	0.10	6.31	0.25	12.12
	1.sSMPABC080	125.25	8.35	498.06	<-NaN	400232.66	0.15	<3.05	0.04	8.19
**TATIA**	1.sSMPABC131	714.24	304.65	1645.57	<-NaN	400232.63	0.31	147.92	3.49	7.69
	1.sSMPABC132	6621.04	107.60	657.50	<-NaN	400232.63	0.21	<3.21	0.13	9.65
	1.sSMPABC112	4450.14	12.47	488.88	<-NaN	400232.63	0.14	3.56	0.03	9.19
**RP1**	1.sSMPABC095	2441.27	112.22	859.80	<-NaN	400232.66	0.35	14.70	0.10	10.04
	1.sSMPABC099	2977.64	2.70	552.70	<-NaN	400232.66	0.19	<3.53	0.07	10.59
	1.sSMPABC106	5879.10	14.53	532.83	<-NaN	400232.66	0.11	<3.24	0.02	10.94
	1.sSMPABC107	2673.22	87.44	614.56	<-NaN	400232.66	0.16	9.55	0.17	9.55
	1.sSMPABC096	1134.26	2.57	551.47	<-NaN	400232.66	0.28	<3.62	0.07	9.29
	1.sSMPABC108	4303.80	754.91	1382.87	<-NaN	400232.66	0.27	7.23	0.88	10.52

Mn55	Fe57	Co59	Ni60	Cu63	Zn66	Ga69	Ge72	As75	Se77	Rb85	Sr88
208.89	<-1.33	3.37	7.46	0.30	1.09	0.72	0.72	<0.227	<0.00	1.10	1268.96
209.36	<-1.32	2.62	7.61	0.48	1.92	5.13	0.79	<0.217	<0.00	4.14	1329.51
210.52	<-0.84	<0.221	8.09	0.22	0.69	0.60	0.54	<0.226	<0.00	0.04	3893.69
241.87	<-0.86	<0.22	7.88	0.34	0.81	0.34	0.52	0.58	<0.00	0.46	1752.21
248.39	<-0.77	<0.21	8.02	0.24	0.81	0.51	0.73	0.25	<0.00	0.01	3464.72
118.53	<-0.80	<0.22	8.10	0.14	0.69	0.11	0.63	0.37	<0.00	<0.0100	3704.77
728.15	<-0.71	<0.23	8.05	0.29	0.70	3.20	0.69	0.91	<0.00	0.06	3842.28
292.56	<-0.74	<0.20	7.70	0.15	1.15	0.37	0.53	<0.191	<0.00	<0.0113	3123.91
670.64	<-0.69	<0.22	8.79	0.32	0.97	1.08	0.69	0.64	<0.00	0.14	2930.33
19.68	<-0.69	<0.23	7.88	0.32	0.76	1.83	0.68	<0.210	<0.00	0.02	4485.18
11.23	<-0.50	<0.26	8.10	0.81	0.98	2.99	0.61	0.25	<0.00	0.42	5498.95
53.80	<-0.51	<0.26	8.87	0.23	1.56	2.31	0.75	0.31	<0.00	0.21	3047.44
73.90	<-0.54	<0.24	7.84	0.20	0.62	3.49	0.59	0.23	<0.00	<0.0148	2350.31
36.88	<-0.67	<0.26	7.59	0.37	8.89	8.27	0.68	0.43	<0.00	0.27	3340.74
28.23	<-0.70	<0.27	8.19	0.38	22.20	1.76	0.60	<0.232	<0.00	<0.0168	2688.58
14.50	<-0.51	<0.25	8.29	0.55	3.88	0.97	0.59	0.36	<0.00	<0.0195	3138.88
3.67	<-0.43	<0.21	8.28	0.16	1.00	21.14	0.55	0.68	<0.00	0.07	4438.85
119.51	<-0.74	<0.28	8.24	1.55	1.75	1.12	0.63	<0.233	<0.00	<0.0204	1683.68
33.43	<-0.53	<0.24	8.50	0.25	7.41	12.05	0.57	1.20	<0.00	0.53	3221.33

Y89	Zr90	Zr91	Nb93	Mo95	Ag107	Cd111	Sn118	Sb121	Cs133	Ba137	La139
2.00	<0.52	<1.05	0.13	0.15	<0.104	<0.095	<0.045	0.03	0.07	4.98	2.70
2.11	3.45	2.28	0.35	0.16	<0.096	<0.091	0.11	0.06	0.24	44.08	2.90
0.61	<0.230	<0.51	0.01	0.13	<0.075	<0.045	<0.042	<0.0118	<0.0080	4.66	0.44
3.80	<0.204	0.56	0.06	0.09	<0.073	<0.043	<0.050	0.01	0.03	2.62	1.12
7.13	<0.185	<0.38	<0.0071	0.07	<0.065	<0.040	<0.042	0.02	<0.0050	4.30	2.79
1.39	<0.171	<0.39	<0.0079	<0.061	0.07	<0.039	<0.043	<0.0120	<0.0058	0.87	0.16
3.29	<0.175	<0.35	<0.0095	0.39	0.22	<0.041	<0.045	0.02	<0.0066	27.10	1.48
3.19	<0.164	<0.35	0.01	0.10	0.10	<0.036	<0.038	<0.0114	<0.0057	2.89	1.15
5.96	<0.164	<0.36	0.01	0.35	0.20	<0.036	<0.048	0.02	0.02	6.76	2.16
5.30	<0.154	<0.35	<0.0055	<0.060	<0.135	<0.040	<0.048	0.02	<0.0074	19.78	2.58
1.65	0.72	0.79	0.08	<0.117	<0.090	0.05	<0.050	0.04	0.04	24.54	1.51
1.18	0.15	<0.33	<0.0087	<0.098	<0.092	<0.033	<0.046	0.02	0.04	18.73	1.43
0.30	<0.128	<0.31	<0.0061	<0.084	<0.089	<0.032	<0.047	<0.0153	0.02	26.42	0.36
1.38	<0.171	<0.38	0.03	<0.105	<0.111	<0.039	0.09	<0.0134	0.04	71.79	0.63
0.96	<0.173	<0.36	0.00	<0.085	<0.114	<0.037	<0.051	<0.0154	0.02	13.83	0.45
0.30	<0.152	<0.31	<0.01	<0.116	<0.103	<0.038	0.05	<0.0131	0.02	8.28	0.24
0.48	0.14	<0.27	0.01	<0.073	0.12	<0.031	<0.038	<0.0108	0.01	171.55	0.33
1.08	<0.175	<0.36	<0.0051	<0.098	<0.124	<0.043	<0.054	<0.0127	0.01	9.72	0.49
0.83	0.21	0.32	0.02	<0.091	0.10	<0.035	<0.045	0.02	0.05	103.35	0.72

Ce140	Pr141	Nd143	Sm147	Eu151	Gd157	Tb159	Dy163	Ho165	Er166	Tm169	Yb172
3.66	0.53	1.92	0.29	0.07	0.41		0.30	0.05	0.09	0.04	0.11
4.46	0.62	2.64	0.30	0.08	0.64		0.20	0.05	0.16	0.04	0.18
0.93	0.08	0.34	0.07	0.02				0.01	0.08	0.02	0.04
0.67	0.20	0.97	0.24	0.05	0.24	0.06	0.34	0.09	0.28	0.03	0.14
3.04	0.67	3.21	0.76	0.15	0.91	0.13	0.76	0.17	0.53	0.07	0.23
0.11	0.04	0.12	0.07	0.01		0.03	0.21	0.02	0.08		0.06
0.99	0.29	1.41	0.41	0.08	0.69	0.08	0.47	0.07	0.26	0.06	0.11
0.56	0.25	1.31	0.29	0.06	0.28	0.04	0.37	0.09	0.16	0.03	0.12
1.05	0.45	2.27	0.50	0.12	0.67	0.08	0.69	0.16	0.54	0.05	0.24
1.80	0.47	2.21	0.36	0.12	0.65	0.11	0.70	0.14	0.45	0.07	0.17
2.17	0.31	1.40	0.21	0.05	0.34	0.03	0.27	0.03	0.10	0.04	0.09
2.10	0.28	1.14	0.17	0.04	0.33		0.17	0.03	0.10		0.05
0.47	0.05	0.26	0.09	0.02	0.16	0.01	<0.118	0.01	0.04	<0.029	<0.047
1.15	0.15	0.38	0.23	0.06	0.56		0.21	0.03		0.03	0.12
1.03	0.10	0.50	0.14	0.05	0.34			0.02	0.09		0.10
0.55	0.07	0.26						0.00			0.05
0.64	0.07	0.32	0.16	0.02	1.61	0.02		0.01	0.04	0.02	0.04
0.79	0.11	0.39	0.13	0.06	0.27	0.03	<0.129	0.03	0.12	<0.034	0.09
1.21	0.15	0.72	0.15	0.03	1.88	<0.0130	<0.100	0.02	0.08	0.02	0.09

Lu175	Hf177	Ta181	W182	Re185	Pb208	Th232	U238	Total REE	Y/Ho	Zn/Al	U/Al
0.02	<0.187	<0.030	0.07	<0.0083	1.15	0.17	0.19	12.19	42.19	16.20	2.80
0.03	<0.180	0.05	0.14	<0.0076	1.09	0.31	0.30	14.41	41.21	6.86	1.07
0.01	<0.083	<0.0194	<0.036	<0.0056	0.41	0.05	0.38	2.63	42.07	221.86	121.28
0.02	<0.076	<0.025	<0.026	<0.0065	0.32	0.02	0.39	8.26	41.76	38.29	18.22
0.05	<0.109	<0.0250	<0.0211	<0.0051	0.67	0.00	0.42	20.60	42.44	1124.13	587.17
	<0.081	<0.026	0.04	<0.0043	0.32	<0.0101	0.19	2.30	58.65	852.40	233.70
0.02	<0.061	<0.032	0.25	<0.0036	0.44	0.01	0.50	9.71	45.19	122.84	88.95
0.01	<0.090	<0.0160	0.03	<0.0042	0.27	0.01	0.22	7.89	37.53	1407.59	268.05
0.03	0.04	<0.0195	0.05	<0.0052	0.59	0.02	0.62	14.95	37.25	128.48	82.25
0.02	<0.064	0.02	0.04	<0.0062	0.38	0.01	0.85	15.14	37.86	905.39	1017.96
	<0.095	0.03	<0.0141	<0.0051	1.54	0.18	0.95	8.20	52.22	32.17	31.18
	<0.067	0.03	<0.0124	<0.0049	1.48	0.11	0.27	7.03	42.60	144.98	25.09
<0.0114	0.06	<0.0184	0.02	<0.0043	0.52	0.01	0.38	1.77	35.90	496.39	303.13
	<0.118	<0.023	0.02	<0.0054	1.02	0.05	0.17	4.90	53.91	792.19	14.70
	<0.106	<0.028	<0.0189	<0.0072	0.61	0.01	0.06	3.79	54.24	82222.22	203.70
	<0.097	<0.0158	<0.0172	<0.0060	0.22	<0.0085	0.14	1.47	61.43	2670.34	92.91
	<0.054	<0.0150	0.02	<0.0033	0.19	0.02	0.24	3.75	48.47	114.36	27.45
<0.0104	<0.124	<0.027	<0.0146	<0.0057	0.31	0.02	0.13	3.58	35.64	6809.34	498.05
0.01	<0.064	<0.0219	<0.021	<0.0048	0.35	0.03	0.33	5.92	38.56	98.16	4.38

V/Al	Ni/Al	Ba/Al	Cu/Al		Y/Ho	Ce/Ce*
17.21	111.15	74.20	4.50		42.19	0.79
13.37	27.20	157.54	1.73		41.21	0.96
30.40	2616.43	1507.12	69.86		42.07	1.71
14.39	372.96	124.01	16.05		41.76	0.50
352.86	11185.50	5997.21	333.33		42.44	0.68
54.61	9963.10	1070.11	177.12		58.65	0.21
77.66	1420.76	4782.92	50.48		45.19	0.52
27.78	9424.72	3537.33	177.48		37.53	0.36
32.98	1164.24	895.36	42.25		37.25	0.37
49.70	9437.13	23688.62	385.63		37.86	0.56
114.56	265.88	805.51	26.56		52.22	0.96
12.27	824.35	1740.71	21.47		42.60	0.92
21.49	6287.09	21186.85	163.59			1.47
8.82	676.35	6397.26	33.15		53.91	0.64
255.56	30333.33	51222.22	1400.00		54.24	1.51
14.25	5705.44	5698.55	377.84		61.43	0.94
19.10	946.94	19619.17	18.18		48.47	1.43
276.26	32062.26	37821.01	6031.13		35.64	0.77
11.66	112.60	1369.04	3.31		38.56	1.20
